# Glucocorticoid receptor modulation decreases ER-positive breast cancer cell proliferation and suppresses wild-type and mutant ER chromatin association

**DOI:** 10.1186/s13058-019-1164-6

**Published:** 2019-07-24

**Authors:** Eva Tonsing-Carter, Kyle M. Hernandez, Caroline R. Kim, Ryan V. Harkless, Alyce Oh, Kathleen R. Bowie, Diana C. West-Szymanski, Mayra A. Betancourt-Ponce, Bradley D. Green, Ricardo R. Lastra, Gini F. Fleming, Sarat Chandarlapaty, Suzanne D. Conzen

**Affiliations:** 1 Department of Medicine, The University of Chicago, Chicago, IL 60637 USA; 2 Center for Research Informatics, The University of Chicago, Chicago, IL 60637 USA; 3 Department of Pediatrics, The University of Chicago, Chicago, IL 60637 USA; 4 Ben May Department for Cancer Research, The University of Chicago, 900 E 57th St, Chicago, IL 60637 USA; 5 Department of Pathology, The University of Chicago, Chicago, IL 60637 USA; 6 Department of Medicine, Memorial Sloan Kettering Cancer Center, New York, NY 10065 USA

**Keywords:** Breast cancer, Estrogen receptor, Glucocorticoid receptor, Mutant activated estrogen receptor, Nuclear receptor crosstalk, Chromatin association, Cyclin D1

## Abstract

**Background:**

Non-ER nuclear receptor activity can alter estrogen receptor (ER) chromatin association and resultant ER-mediated transcription. Consistent with GR modulation of ER activity, high tumor glucocorticoid receptor (GR) expression correlates with improved relapse-free survival in ER+ breast cancer (BC) patients.

**Methods:**

In vitro cell proliferation assays were used to assess ER-mediated BC cell proliferation following GR modulation. ER chromatin association following ER/GR co-liganding was measured using global ChIP sequencing and directed ChIP analysis of proliferative gene enhancers.

**Results:**

We found that GR liganding with either a pure agonist or a selective GR modulator (SGRM) slowed estradiol (E2)-mediated proliferation in ER+ BC models. SGRMs that antagonized transcription of GR-unique genes both promoted GR chromatin association and inhibited ER chromatin localization at common DNA enhancer sites. Gene expression analysis revealed that ER and GR co-activation decreased proliferative gene activation (compared to ER activation alone), specifically reducing *CCND1*, *CDK2*, and *CDK6* gene expression. We also found that ligand-dependent GR occupancy of common ER-bound enhancer regions suppressed both wild-type and mutant ER chromatin association and decreased corresponding gene expression. In vivo, treatment with structurally diverse SGRMs also reduced MCF-7 Y537S ER-expressing BC xenograft growth.

**Conclusion:**

These studies demonstrate that liganded GR can suppress ER chromatin occupancy at shared ER-regulated enhancers, including *CCND1* (*Cyclin D1*), regardless of whether the ligand is a classic GR agonist or antagonist. Resulting GR-mediated suppression of ER+ BC proliferative gene expression and cell division suggests that SGRMs could decrease ER-driven gene expression.

**Electronic supplementary material:**

The online version of this article (10.1186/s13058-019-1164-6) contains supplementary material, which is available to authorized users.

## Background

In breast cancer (BC), functional interaction between estrogen receptor (ER) and other nuclear receptors (NRs) has recently been recognized to play an important role in ER-mediated tumor cell proliferation [1–3]. For example, progesterone (PR) [1, 2], androgen (AR) [4, 5], and glucocorticoid receptor (GR) [3, 6–9] activation all result in modification of ER-mediated gene expression. Now that it is appreciated that NR crosstalk with ER occurs in ER+ BC and endometrial cancer [10, 11], we have a mechanistic framework for understanding the improved prognosis of ER+ BCs with high GR or PR expression [12, 13]. Previously, it was noted that ER binding to a subset of ER target gene enhancer regions could be inhibited by GR agonism with dexamethasone (Dex) [7]. This finding was consistent with highly dynamic chromatin-specific GR/ER crosstalk in model systems [3, 6–9, 14, 15].

Here, we hypothesized that GR liganding might specifically inhibit ER-mediated BC cell proliferation through a dynamic displacement of ER at key pro-proliferative gene regulatory regions (*CCND1*, *CDK2*, and *CDK6*). We further hypothesized that this effect could occur with either a pure GR agonist or a selective GR modulator (SGRM), because both ligands can drive GR to DNA regulatory regions. Indeed, we found that either Dex or SGRMs decreased activated ER occupancy at several enhancers and that this displacement was associated with decreased corresponding proliferative gene expression. At early time points (15 and 30 min), newly ligand-bound GR associated with the *CCND1*, *CDK2*, and *CDK6* enhancer regions normally targeted by ER. By 60 min, activated ER chromatin association was relatively suppressed with concomitant GR liganding, suggesting a mutually exclusive GR versus ER chromatin association at these enhancers. The reduction of ER chromatin occupancy was accompanied by a decrease in subsequent expression of targeted pro-proliferative genes and also decreased ER-driven cell proliferation. Findings were similar with wild-type (WT) ER+ MCF-7 cells or cells expressing a mutant (Y537S) constitutively active ER—both demonstrated GR-activation displaced WT or Y537S ER from *CCND1* and *CDK2* enhancers. These findings underscore the important role of GR/ER crosstalk in human BC and suggest that either GR agonists or antagonists can modulate GR chromatin binding so as to result in similar anti-proliferative effects with respect to ER-mediated BC biology.

## Materials and methods

### Cells and cell culture

MCF-7 and T-47D cells were purchased from ATCC and cultured in DMEM supplemented with 10% FBS (Gemini Bio-Products, West Sacramento, CA) and 1% penicillin/streptomycin (Invitrogen, Waltham, MA) at 37 °C and 5% CO_2_. MCF-7 HA-WT, HA-Y537S, and HA-D538G cells were a kind gift of S. Chandarlapaty (MSKCC) and were cultured in DMEM phenol-red free supplemented with 5% FBS, 1% Pen/Strep (Invitrogen, Waltham MA), 100 μg/mL Geneticin (Gibco, Gaithersburg, MD), and 100 μg/mL hygromycin B (Gibco, Gaithersburg, MD) at 37 °C and 5% CO_2_ [16]. For MCF-7 HA-WT, HA-Y537S, and HA-D538G cells, MCF7 Tet-ON cells (Clontech, Mountain View, CA) were infected with retroviral vectors containing either doxycycline-inducible HA-tagged ER wild-type (WT) or Y537S or D538G mutants. For forty-eight-hour post-infection, the infected cells were selected with 500 μg/mL of hygromycin for a period of 14 days, in which afterwards, hygromycin concentration was lowered to 100 μg/mL for regular passaging of the stable cell lines [16, 17]. For all experiments, cells were seeded in normal growth medium. When cells reached ~ 60–80% confluence, they were placed in 2.5% charcoal-stripped serum (CSS) in phenol-red free DMEM for 48–72 h prior to hormone treatment. For hormone treatments, cells were treated with vehicle (Veh, ETOH), 10 nM E2 (Sigma-Aldrich, St. Louis, MO), 100 nM dexamethasone (Dex, Sigma-Aldrich, St. Louis, MO), 1 μM CORT125134 (C134, Corcept Therapeutics, Menlo Park, CA), 1 μM CORT118335 (C335, Corcept Therapeutics, Menlo Park, CA), or 1 μM CORT108297 (C297, Corcept Therapeutics, Menlo Park, CA). Final ETOH concentration did not exceed 0.2%. For HA-tagged cells, expression of the HA-tagged wild type or Y537S or D538G was induced following 0.5 μg/mL doxycycline (Sigma-Aldrich, St. Louis, MO) when cells were placed in CSS containing media. Cells regularly tested negative for mycoplasma using the Universal Mycoplasma Detection Kit (ATCC, Manassas, VA).

### Western blot

Cells were cultured in phenol red-free DMEM supplemented with 2.5% CSS and 1% Pen/Strep (Invitrogen, Waltham, MA) for 48 h, and cells were lysed with RIPA lysis buffer with phosphatase and protease inhibitors (Roche Diagnostics USA, Indianapolis, IN). Protein was quantified using Pierce BCA Protein Assay (Thermo Scientific, Waltham, MA) per manufacturer’s instructions. Protein (50 μg) was loaded per sample and resolved with SDS-PAGE. Membranes were blocked with 5% milk (Roche Diagnostics USA, Indianapolis, IN) or 5% BSA (Sigma-Aldrich, St. Louis, MO) in TBST. Membranes were immunoblotted with anti-GR (1:500, 41/GR, BD Biosciences, San Jose, CA), anti-ER (1:500, F10, Santa Cruz Biotechnology, Dallas, TX), anti-PR (1:1000, D8Q2J, Cell Signaling, Danvers, MA), anti-HA (1:1000, C29F4, Cell Signaling, Danvers, MA), anti-Cyclin D1 (1:100,000, EPR2241, Abcam, Cambridge, MA), anti-β-actin (1:1000, 8H10D10, Cell Signaling, Danvers, MA), or α-tubulin (1:5000, DM1A, Millipore, Burlington, MA). Densitometry analysis was performed using ImageJ version 1.52a. The intensity of Cyclin D1 and β-actin bands were quantified, and results are reported as a ratio of cyclin D1 band intensity/β-actin band intensity for each treatment condition.

### Longitudinal cell proliferation

MCF-7 and T-47D cells (2.5 × 10^4^) were seeded in 12-well plates. Cells were cultured in phenol red-free DMEM supplemented with 2.5% CSS and 1% Pen/Strep for 48 h and then treated with Veh (ETOH), 100 nM Dex/V, 10 nM E2/V, Dex/E2, 1 μM C335/E2, 1 μM C134/E2, or 1 μM C297/E2. Cells were harvested, and total live and dead cells were counted 0–8 days post-treatment using trypan blue exclusion. Experiments were repeated *n* = 3 times.

### Cell cycle analysis

MCF-7 cells were seeded in 10-cm dishes. Cells were cultured in phenol red-free DMEM supplemented with 2.5% CSS and 1% Pen/Strep for 72 h. Cells were synchronized for 72 h in 0% CSS containing medium. Cells were treated with Veh (ETOH), 10 nM E2/V, 100 nM Dex/E2, 1 μM C335/E2, or 1 μM C134/E2 and fixed with 70% ETOH. Cells were stained with 0.02 mg/mL propidium iodide and examined by FACS. Cell cycle populations were determined using FlowJo Software (Ashland, OR).

### Methylene blue

The methylene blue proliferation assay was derived from Oliver and colleagues [18]. MCF-7 HA-tagged wild-type (WT), Y537S, or D538G cells were seeded in 96-well plates with 2.5% CSS containing medium and treated with vehicle (ETOH), 100 nM Dex/V, 10 nM E2/V, Dex/E2, 1 μM C335/E2, or 1 μM C134/E2 in triplicate or sextuplicate. Cells were fixed with methanol and stained with 0.05% methylene blue as described in [19].

### Microarray

MCF-7 cells were seeded in 10-cm dishes. Cells were cultured in phenol red-free DMEM supplemented with 2.5% CSS and 1% Pen/Strep for 48 h and treated with Veh (ETOH), 10 nM E2/V, 100 nM Dex/E2 for 4 h. Total RNA was harvested with RNeasy Kit (Qiagen, Germantown, MD) and examined by Affymetrix HG U133+2.0 microarray. The arrays were processed in R; and fold-changes were determined using a 1.3-fold cutoff (see Additional files 1 and 2) and examined by Ingenuity Pathway Analysis (IPA, Qiagen, Germantown, MD).

### qRT-PCR

MCF-7 and T-47D cells were seeded in 10-cm dishes. Cells were cultured in phenol red-free DMEM supplemented with 2.5% CSS and 1% Pen/Strep for 48 h and treated with Veh (ETOH), 10 nM E2/V, 100 nM Dex/E2, 1 μM C335/E2, or 1 μM C134/E2. Cells were harvested at 4 and 24 h post-treatment, and total RNA was isolated using RNeasy Mini Kit. cDNA was synthesized using qScript cDNA SuperMix (Quanta Biosciences, Beverly, MA). qRT-PCR was carried out using PerfeCTa SYBR Green FastMix (Quanta Biosciences, Beverly, MA). RPLP0 transcript was used as an internal control. Experiments were repeated *n* = 3 times with triplicate or quadruplicate wells for each treatment group within each experiment.

### ChIP-seq

MCF-7 cells were incubated in phenol red-free DMEM containing 2.5% CSS for 4 days total (with a media change after 48 h) and treated with either ETOH (60 min), 100 nM dexamethasone (60 min), or 100 nM E2 (75 min) or pretreated with E2 for 15 min, followed by co-treatment with dexamethasone (i.e., 75 min E2 and 60 min dexamethasone). These hormone concentrations were used previously in GR ChIP-seq experiments [9]. DNA and associated proteins were crosslinked with 1% formaldehyde, and lysates were sonicated and immunoprecipitated as described previously [9]. ChIP experiments were conducted using the ChIP Assay Kit and the manufacturer’s protocol (EMD Millipore). Three-microgram ChIP-grade anti-ER HC-20 (sc-543x, Santa Cruz Biotechnology, Dallas, TX or sc-8002x, Santa Cruz, Dallas, TX) was used for immunoprecipitation. We also used normal rabbit IgG (#2729, Cell Signaling, Danvers, MA) as a negative control. Eluted ChIP DNA was purified using the PCR Purification Kit (Qiagen, Beverly, MA). ER ChIP-seq was performed on the Illumina HiSeq platform, generating raw reads for analysis (see Additional file 1). The sequence alignment and identification of peaks is described briefly. Sequence quality was assessed and aligned to the human genome (version hg19), and peaks were detected using MACS2 v2.1.1.20160309 [20].

### Directed ChIP-PCR

For directed anti-GR or anti-ER ChIP experiments, MCF-7 cells were treated as described above. MCF-7 HA-tagged Y537S cells were incubated in phenol-red free medium with 2.5% CSS and 0.5 μg/mL doxycycline for at least 48 h and treated with vehicle (ETOH), 100 nM Dex, 1 μM C134, or 1 μM C335 for 15, 30, or 60 min. Cells were lysed as described above, and 3 μg ChIP-grade rabbit monoclonal anti-GR (GRXP, #3660, Cell Signaling, Danvers, MA), anti-ER (F10, sc-8002x, Santa Cruz Biotechnology, Dallas, TX), and anti-HA (F-7, sc-7392x, Santa Cruz Biotechnology, Dallas, TX) were used for immunoprecipitation. Normal rabbit IgG (#2729, Cell Signaling, Danvers, MA) or normal mouse IgG2a (E5Y6Q, #61656, Cell Signaling, Danvers, MA) was used for negative controls. Eluted ChIP DNA was purified using the PCR Purification Kit (Qiagen, Beverly, MA) and quantified by Qubit. GR and ER ChIP-seq peaks for specific genes (*CCND1*, *CDK2*, and *CDK6*) were visualized using the Integrative Genomics Viewer (The Broad Institute, Cambridge, MA). Primers for enh2 (*CCND1*) were previously published [21] and primers for *CDK2* (CDK2-F 5′-CAGACTGCCTTCTATCCCAGA-3′; CDK2-R 5′-AGTGGCTTCTGGGAAAGGAA-3′) and *CDK6* (CDK6-F: 5′-AGCTTAGCGCCTGAGAGATG; CDK6-R: CAGAGGCATCTGTTCTGCAA) putative enhancers were designed using Primer3 [22]. qPCR was carried out using PerfeCTa SYBR Green FastMix (Quanta Biosciences, Beverly, MA) and fold changes were calculated relative to vehicle-treated cells. *FKBP5* [7] and *TFF1* [7] enrichment was used as a control for GR or ER ChIP, respectively.

### Animal studies

All studies were carried out in accordance with and approval of the Institutional Animal Care and Use Committee of The University of Chicago and the Guide for the Care and Use of Laboratory Animals. One week prior to cell implantation, female mice between 5 and 7 weeks of age began a diet of doxycycline-containing chow (TD.01306, Envigo, NJ). MCF-7 HA-Y537S cells (10 × 10^6^) were implanted into the 2nd thoracic mammary fat pad of *SCID* mice (Taconic, Rensselaer, NY) and allowed to grow. Mammary tumors were measured twice weekly by caliper, and tumor volume was calculated as described previously [23]. Mice were randomized into treatment groups, and when tumors reached ~ 200mm^3^, mice were treated with vehicle (1 ETOH:9 sesame oil, Spectrum, Gardena, CA), 20 mg/kg C134, or 20 mg/kg C335 intraperitoneally (i.p.) thrice weekly until the end of the study. Tumors were snap frozen for RNA analysis or fixed in 10% neutral buffered formalin for immunohistochemistry.

### Xenograft qRT-PCR

Xenograft tumors were homogenized in Blue Bullet Blender tubes (Next Advantage, Troy, NJ) with Buffer RLT (Qiagen, Germantown, MD), and RNA was extracted per manufacturer’s instructions. qRT-PCR was performed as described above.

### Immunohistochemistry (IHC)

For MCF-7 Y537S tumor xenograft IHC analysis, tissues were fixed in 10% neutral-buffered formalin and embedded in paraffin. Sections (5 μm) were stained with anti-Ki67 (1:300, SP6, Thermo Scientific, Waltham, MA), anti-Cyclin D1 (1:100, EPR2241, Abcam, Cambridge, MA), or anti-ER (1:50, RM-9101, Thermo-Fisher, Waltham, MA). Anti-Ki-67, anti-Cyclin D1, and anti-ER IHC immunoreactivity were scored by our pathologist (RL) blinded to the treatment conditions. Anti-Ki-67 IHC staining was evaluated by counting the percentage of positive cells (any intensity) in a full tumor section. Anti-ER IHC staining was evaluated by an H-score, which accounts for intensity and percent positivity and yields a score of 0–300 [24]. For anti-Cyclin D1 staining assessment, because the percentage of positive tumor cells was uniform > 95%, slides were scored as a binary high- versus medium/low-intensity staining and significance was determined by Fisher’s exact test. Differences in staining intensity were considered significant if *p* < 0.05. Data are presented as mean ± standard deviation (SD). Representative images were taken using a Nikon Eclipse Ti2 microscope at × 200 total magnification using identical settings for each slide.

## Results

### GR modulation decreases ER-mediated breast cancer cell proliferation

**Fig. 1:**
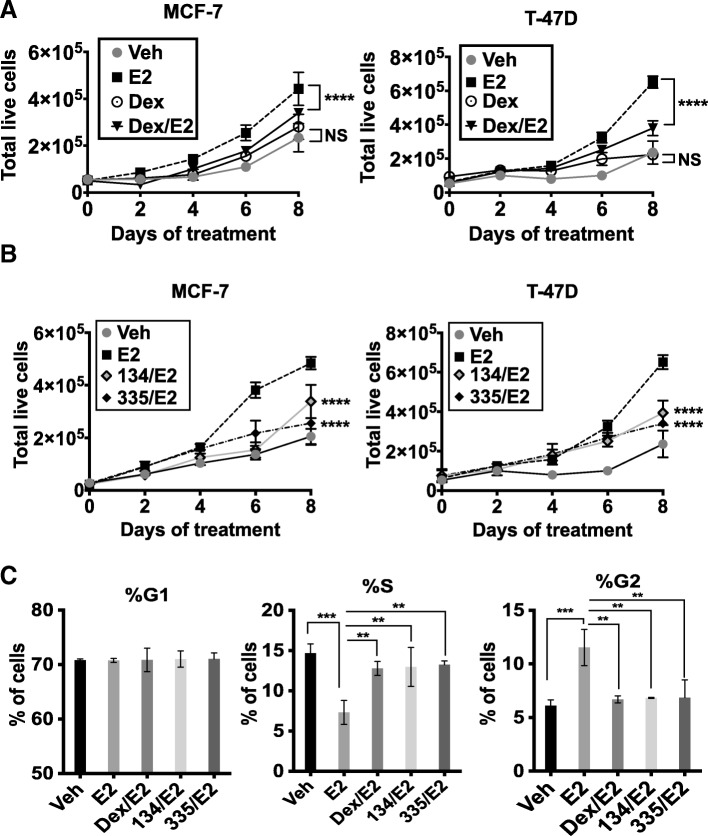
Treatment with either Dex or a selective GR modulator (SGRM) suppresses ER-mediated cell proliferation. **a** MCF-7 and T-47D cells were treated for 8 days with either vehicle (Veh, ETOH), dexamethasone (Dex, 100 nM), estradiol (E2, 10 nM), or Dex/E2 and total live cells counted. E2 treatment-alone (ER activation) significantly increased cell proliferation compared to either Veh or Dex-alone. Dex/E2 significantly reduced E2-mediated proliferation. There was no difference between Dex and Veh treatment (****p < 0.0001, vs. Veh, two-way ANOVA, Holm-Sidak post hoc test; NS, not significant, *n* = 4 per group, ± SD). **b** MCF-7 and T-47D cells were treated with vehicle (ETOH), 10 nM E2, E2/1 μM C134 or E2/1 μM C335 and total live cells counted. GR-selective liganding with C134 or C335 significantly reduced E2-mediated proliferation (****p < 0.0001, vs. E2-alone, two-way ANOVA, Holm-Sidak post hoc test, *n* = 4 per group, ±SD). **c** FACS cell cycle analyses were performed and revealed GR-selective liganding significantly inhibited G2 cell cycle progression compared to E2-alone treated cells (**p < 0.01, ***p < 0.001 one-way ANOVA, Tukey’s post hoc test, *n* = 3 per group, ±SD)

Previous observations suggested that GR agonists inhibit ER-driven BC cell proliferation [3, 7, 9, 25, 26]. To validate these findings in our GR+/ER+ BC cell models (Additional file 3: Figure S1A), we treated GR+/ER+ MCF-7 and T-47D cells with vehicle (ETOH), dexamethasone (Dex, 100 nM), estradiol (E2, 10 nM), or Dex/E2 (Fig. 1a). Following co-treatment with Dex/E2, we indeed observed a significant reduction in cell proliferation compared to E2 treatment alone. However, GR liganding with the synthetic glucocorticoid Dex in the absence of E2 was not growth suppressive suggesting that GR specifically reduces E2-mediated proliferation (Fig. 1a).

Because long-term glucocorticoid treatment is not well-tolerated in patients due to metabolic side effects [27], we tested the effect of novel selective GR modulators (SGRMs) on ER-mediated BC cell proliferation. GR liganding with Dex results in an upregulation of canonical GR target genes (e.g., *FKBP5*) while the SGRMs C134 and C335 are GR antagonists with respect to canonical GR target gene expression (Additional file 3: Figure S1B). Surprisingly, we found that SGRMs [28–30], similarly to Dex, resulted in significant inhibition of E2-mediated proliferation (Fig. 1b), suggesting that specific GR ligand binding per se results in slowed E2-mediated proliferation. The reduced cell number observed following several days of highly GR-selective liganding was not accompanied by increased cell death (Additional file 3: Figure S1C). However, cell cycle analysis showed slowing of progression from S to G2/M at 18 h in MCF-7 cells as reflected by increased S-phase accumulation (Fig. 1c). In the GR-negative T47D-Y cell line [31] (Additional file 4: Figure S2A), we observed that neither Dex nor the SGRMs CORT108297 [32] or C335 decreased E2-driven cell proliferation (Additional file 4: Figure S2B), suggesting a GR-specific mechanism of action. Furthermore, a radioactive E2 competition assay using recombinant ER ligand-binding domain (LBD) showed no radioactive E2 displacement by Dex, C134, or C335 (Additional file 4: Figure S2C), confirming that SGRMs are not interacting directly with ER LBD. Taken together, these data suggest that either a pure GR agonist (Dex) or a SGRM can inhibit ER-driven BC cell proliferation through a GR-mediated effect on ER activity.

### GR modulation decreases ER-mediated proliferative gene expression

**Fig. 2:**
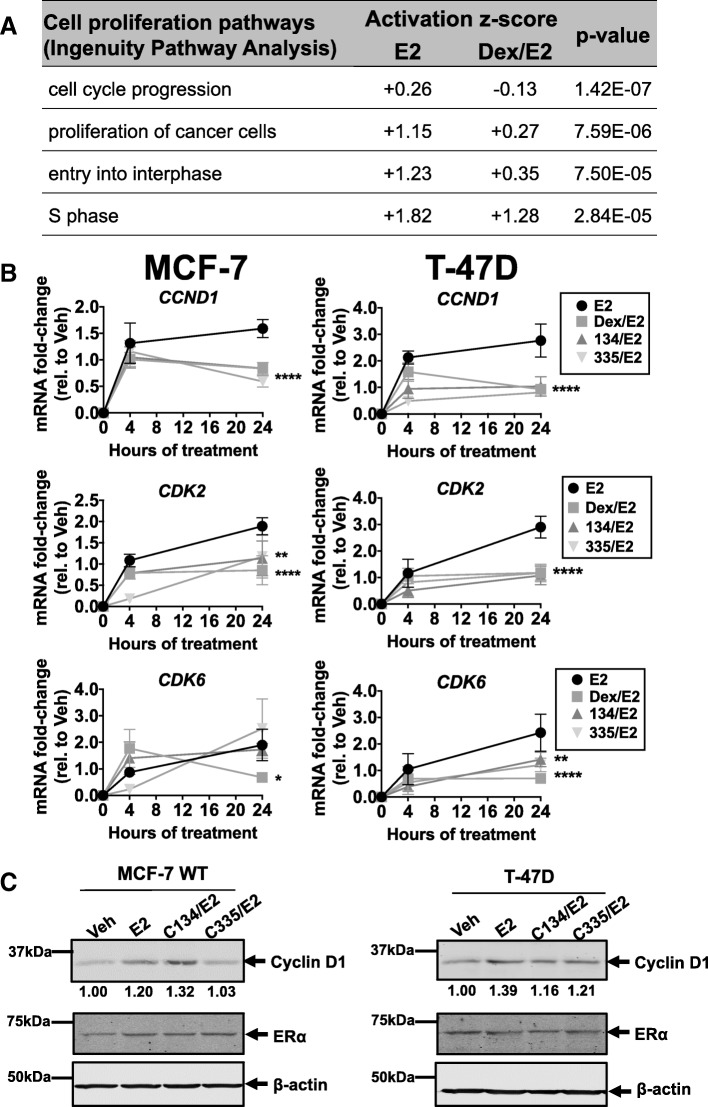
GR liganding inhibits ER-mediated proliferative gene expression. **a** MCF-7 cells were treated with vehicle (ETOH), 100 nM Dex, 10 nM E2, or Dex/E2 for 4 h and global gene expression was evaluated by microarray followed by Ingenuity Pathway Analysis (IPA). Proliferation and cell cycle pathways were identified as those with the lowest *p* values; pathway activation *z*-score is also shown. Dex/E2 treatment consistently demonstrated relatively decreased gene expression pathway activation (*z*-score) compared to E2-alone. **b** Genes common to cell proliferation pathways in 2A (*CCND1*, *CDK2*, and *CDK6*) were assessed by qRT-PCR in independent experiments. All genes demonstrated reduced gene expression by 24 h following Dex treatment; with the exception for *CDK6* in MCF-7 cells, all genes demonstrated significant reduction in gene expression following SGRM treatment (*p < 0.05, **p < 0.01, ****p < 0.0001, two-way ANOVA, Tukey’s post hoc test, *n* = 3, ±SD). C) MCF-7 and T-47D wild-type ER+ cells were treated with vehicle (Veh, ETOH), 10 nM E2, 1 μM C134/E2 (134/E2), or 1 μM C335/E2 (335/E2) for 3 days. Cyclin D1, ERα, and β-actin were immunoblotted. E2 treatment led to an increase in Cyclin D1 protein expression, while C335 led to relatively decreased Cyclin D1 protein expression in MCF-7 cells. C134 led to a more pronounced decrease in Cyclin D1 protein in T-47D cells. Densitometry analysis is shown below the Cyclin D1 bands (relative to Veh). ERα protein expression was unchanged following combined E2/SGRM treatment when compared to either E2-alone or vehicle (Veh)

To begin to determine how GR inhibits ER-mediated cell proliferation, we examined global gene expression in MCF-7 cells following E2, Dex, or E2/Dex co-treatment. Using Ingenuity Pathway Analysis (IPA), we found proliferative gene expression pathways to be significantly activated by E2; this activation was decreased by Dex co-treatment (Fig. 2a). Within these proliferation pathways, we consistently found three common E2-induced cell cycle genes (*CCND1*, *CDK2*, and *CDK6*). Moreover, the mRNA expression for these three ER-target genes was also consistently decreased by treatment with Dex or a SGRM. For example, in MCF-7 cells, we found that E2-induced *CCND1* and *CDK2* gene expression was significantly suppressed at 24 h following treatment with Dex, C134, or C335 (Fig. 2b, left panel). In T-47D cells, GR-selective ligands similarly resulted in suppressed expression of all three cell cycle genes at 24 h (Fig. 2b, right panel). Cyclin D1 protein expression by densitometry was also measured in Western blotting and found to be reduced by the addition of C335 (1.03 versus 1.20 for E2 alone, Fig. 2c). C134 addition did not show uniform reduction of Cyclin D1 amount. In T47-D cells, Cyclin D1 protein expression following the addition of either C134 (1.16) or C335 (1.21) was lower than Cyclin D1 expression after treatment with E2 alone (1.39, Fig. 2c, Western images representative of three independent experiments). Given the biological importance of *CCND1*, *CDK2*, and *CDK6* in ER+ BC cell proliferation [33–35], we next focused on examining ER-associated enhancer regions of these three genes in the context of GR modulation by SGRMs [21, 36, 37].

### GR modulation suppresses ER chromatin association at *CCND1*, *CDK2*, and *CDK6* enhancer regions

**Fig. 3:**
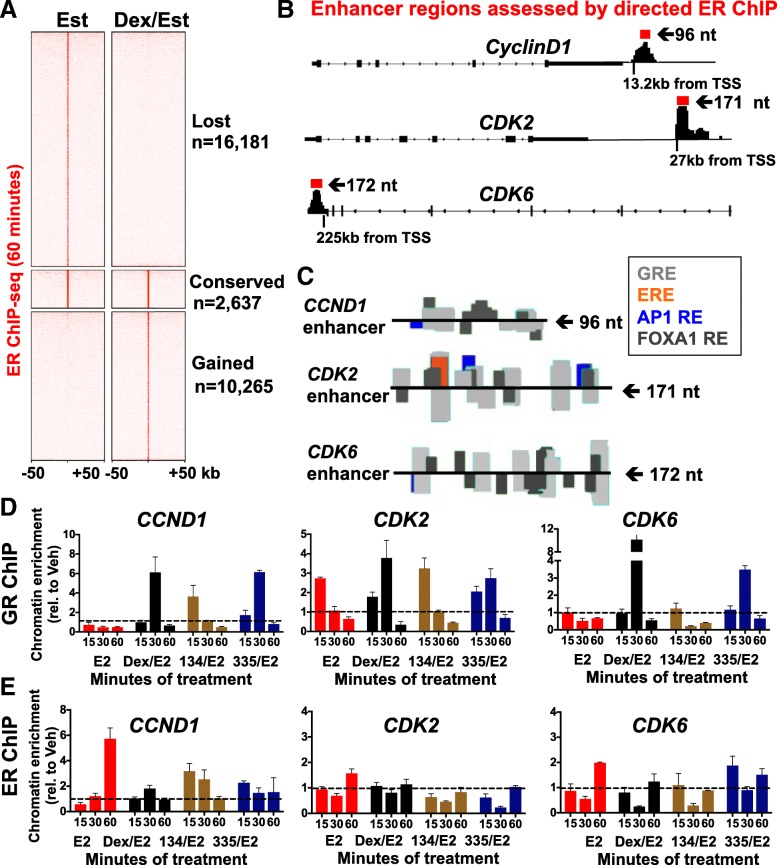
GR liganding alters genome-wide ER chromatin association including disruption of ER chromatin occupancy at *CCND1*, *CDK2*, and *CDK6* enhancer regions. **a** MCF-7 cells were treated with vehicle (ETOH), 100 nM Dex, 100 nM E2, or co-treated with Dex/E2 followed by ER ChIP-seq. There were *n* = 16,181 ER peaks lost and 10,265 ER peaks gained with Dex/E2 co-treatment, while only 2637 ER peaks were conserved when compared to ER peaks following E2-treatment alone. **b** E2-treated cells revealed ER peaks in previously established ER enhancer regions of *CCND1* [21], *CDK2* [36], and *CDK6* [36] as indicated in red. **c** Motif analyses identified a predominance of FOXA1 (dark gray) response elements (REs) and GREs (light gray) in these ER enhancer regions. **d** Directed GR ChIP of MCF-7 cells treated with GR ligands for either 15, 30, or 60 min revealed consistent GR occupancy at known ER enhancer regions (*CCND1*, *CDK2*, and *CDK6*) following GR ligand treatment. **e** Following directed ER ChIP, ER enrichment at the *CCND1*, *CDK2*, and *CDK6* enhancer sites was uniformly suppressed with the addition of GR ligands

It has been reported that GR activation with Dex can cause chromatin remodeling resulting in differential ER chromatin association [8, 9]. We therefore determined whether in our model system GR and ER co-activation caused significant ER chromatin remodeling. Indeed, we observed both ER chromatin loss (*n* = 16,181 peaks) as well as gain of new sites (*n* = 10,265 peaks) at 60 min following GR liganding with only a relatively small number of ER peaks conserved (*n* = 2637) (Fig. 3a). This suggests rapid and highly dynamic global changes in ER association with DNA following GR liganding as previously reported [8, 9]. We next examined whether GR liganding influenced ER-chromatin association at specific enhancers for *CCND1*, *CDK2*, and *CDK6* that had been previously identified using E2-activated ER ChIP [21] and enhancer (e)-RNA detection [36, 37], and further confirmed in our experiments at 60 min of E2 treatment (Fig. 3b). Motif analysis (Possum, https://zlab.bu.edu/~mfrith/possum/) of these three enhancer regions was also performed (Fig. 3c). The *CCND1* enhancer (enhancer 2, enh2) demonstrated a previously known critical FOXA1 site [21] as well as several GR response elements (GREs) and AP1 REs, but no estrogen response elements (EREs) (Fig. 3c). Similarly, for *CDK2* and *CDK6* enhancer regions previously demonstrated to associate with activated ER, there were several GREs, FOXA1 REs, AP1 REs, and only a single ERE found in the *CDK2* enhancer. These analyses are consistent with the current model of ER predominantly binding to chromatin indirectly via cooperative transcription factors, e.g. FOXA1, to regulate enhancer activity [38].

To determine whether ER-chromatin association is altered following GR liganding at enhancer regions for *CCND1* [21], *CDK2* [36], and *CDK6* [36], we performed directed ER and GR ChIP. For the *CCND1* enhancer [21], GR did not bind significantly in the absence of a GR ligand (Fig. 3d, top-left panel). However, in the context of ER activation, GR associated with chromatin as early as 15 min following C134 treatment and at 30 min following Dex or C335 treatment (Fig. 3d, left panel). In parallel, directed ER ChIP experiments revealed significantly increased ER bound to this *CCND1* enhancer region at 60 min following E2 alone [21, 38] (Fig. 3e, left panel). However, ER binding was reduced in the presence of co-liganded GR (Fig. 3e, left panel). These findings suggest that GR chromatin association inhibits activated ER binding to the *CCND1* enhancer. We next evaluated GR chromatin binding at the *CDK2* enhancer (27 kb downstream of the TSS). In this case, GR association increased transiently at 15 min following E2 stimulation (Fig. 3d, middle panel), but was not found at later time points. As with the *CCND1* enhancer, GR association following the addition of C134 peaked at 15 min (Fig. 3d, middle graph) and at 30 min following Dex or C335. As with *CCND1*, ER association with the *CDK2* enhancer peaked at 60 min with E2 alone, but was relatively reduced in the presence of GR liganding (Fig. 3e, middle panel). Finally, we found that GR association with the known *CDK6* enhancer (225 kb downstream from the TSS) showed a similar pattern in the context of ER activation; GR association peaked at 15 min following C134 treatment and at 30 min following Dex or C335 treatment (Fig. 3d, right panel). ER binding to the *CDK6* enhancer was similarly strongest without GR liganding (Fig. 3e, right panel). Interestingly, we noted that C134 induced more rapid maximal GR chromatin association (15 min) compared with Dex or C335 (30 min) at the same compound concentrations (Fig. 3d, right panel), suggesting that different GR ligands produce similar anti-proliferative phenotypes with slightly different kinetics. In summary, for these three pro-proliferative gene enhancer regions, GR chromatin binding induces relatively suppressed ER chromatin occupancy and is associated with subsequently decreased ER-mediated *CCND1*, *CDK2*, and *CDK6* gene expression.

### GR modulation inhibits constitutively-active mutant (Y537S) ER-associated cell proliferation

**Fig. 4:**
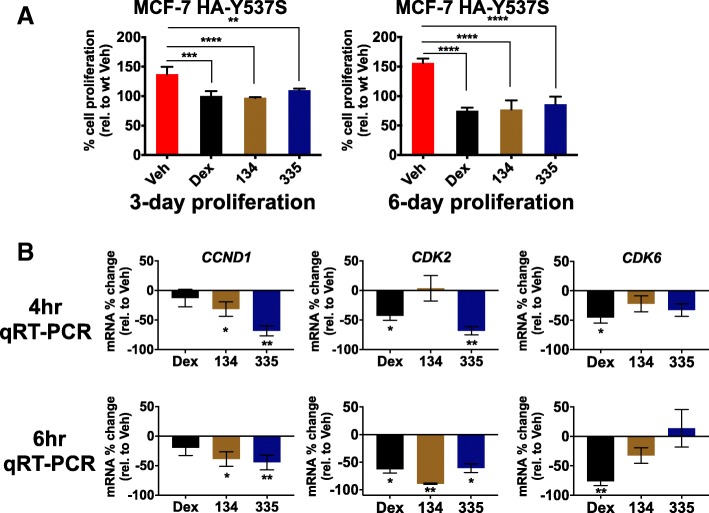
GR activation inhibits constitutively-active Y537S ER-mediated proliferation and ER chromatin association. MCF-7 HA-tagged Y537S cells were pre-treated for 48 h with 0.5 μg/mL doxycycline to induce the expression of HA-tagged Y537S ER. **a** Cells were treated with GR-selective ligands for 3 and 6 days. Dex, C134, and C335 significantly inhibited mutant Y537S ER-mediated proliferation (**p < 0.01, ***p < 0.001, ****p < 0.0001, two-way ANOVA, Tukey’s post hoc test, *n* = 3, ±SD). B) Cells were treated with GR-selective ligands for 4 or 6 h, and *CCND1*, *CDK2*, and *CDK6* gene expression was reduced following GR liganding with Dex, C134, or C335 (*p < 0.05, **p < 0.01, ****p < 0.0001, two-way ANOVA, Tukey’s post hoc test, *n* = 3, ±SD)

Activating ER mutations are found in up to 40% of ER+ BCs that develop resistance to aromatase inhibitor therapy; ER Y537S and D538G are the most commonly observed [16, 39, 40]. Having discovered that GR inhibited estradiol-activated gene expression and proliferation in wild-type ER+ BC cells, we next examined whether GR similarly inhibited constitutively-active mutant ER activation of *CCND1*, *CDK2*, and *CDK6*. Several labs recently reported that both E2-activated wild-type and non-liganded mutant ER target many of the same enhancer regions [39, 41, 42]. We therefore hypothesized that GR modulation could suppress mutant ER transcriptional activity and resultant cell proliferation. Mutant ER (D538G and Y537S)-expressing MCF-7 cells (Additional file 5: Figure S3A) demonstrated significantly increased proliferation compared to wild-type ER+ MCF-7 cells (Additional file 5: Figure S3B) [16, 17]. MCF-7 HA-WT cells showed a significant increase in proliferation following E2 treatment and a significant reduction of E2-mediated proliferation following GR liganding with Dex or SGRMs (Additional file 5: Figure S3C, left panel). We then examined proliferation of MCF-7 cells expressing Y537S or D538G ER following GR-selective liganding and found a relative reduction in cell proliferation (HA-Y537S, Fig. 4A and HA-D538G, Additional file 5: Figure S3C, right panel). We also identified a significant decrease in *CCND1* gene expression following C134 or C335 treatment at 4 and 6 h in MCF-7 HA-Y537S cells (Fig. 4b, left panels). *CDK2* gene expression was significantly decreased following C335 at 4 h and all three ligands at 6 h (Fig. 4b, middle panels). For *CDK6*, gene expression was significantly reduced following Dex treatment at both 4 and 6 h (Fig. 4b, right panels).

For MCF-7 HA-D538G cells, *CCND1* steady-state mRNA was also significantly reduced following Dex and C134 treatment at 4 h (Additional file 5: Figure S3D, left panel). Similarly, *CDK2* steady-state mRNA expression was significantly decreased following all three ligands at 6 h (Additional file 5: Figure S3D, middle panel). CDK6 steady-state mRNA expression was only significantly reduced following C134 treatment in the MCF-7 HA-D538G cells (Additional file 5: Figure S3D, right panel) demonstrating target gene-specific variability of SGRM activity with respect to the D538G ER mutation.

**Fig. 5:**
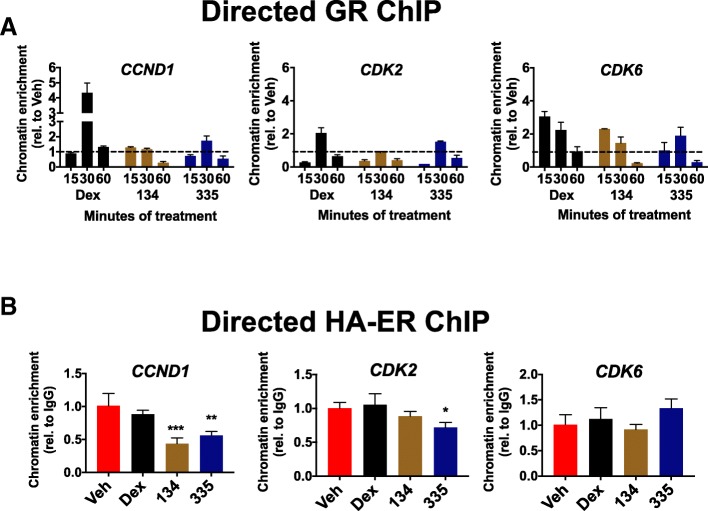
SGRM-mediated GR chromatin association also results in reduced Y537S mutant ER chromatin association at the *CCND1* enhancer region. MCF-7 HA-tagged Y537S cells were pre-treated for 48 h with 0.5 μg/mL doxycycline to induce the expression of HA-tagged Y537S ER and treated with vehicle (Veh, ETOH), 100 nM dexamethasone (Dex), 1 μM C134 (134), or 1 μM C335 (335) for 60 min. **a** GR ChIP analysis in HA-Y537S mutant cells revealed consistent GR occupancy at known ER enhancer regions (*CCND1*, *CDK2*, and *CDK6*) following either Dex or SGRM treatment. **b** HA-ER Y537S enrichment was suppressed with C335 and C134 at 60 min at the *CCND1* and with C335 at the *CDK2* enhancers (***p < 0.001, **p < 0.01, *p < 0.05 vs. Veh, one-way ANOVA, Dunnett’s post hoc test, *n* = 3 ±SD)

We confirmed robust Y537S ER chromatin association at the same *CCND1*, *CDK2*, and *CDK6* enhancer regions previously identified with wild-type ER ChIP (Fig. 3b and Additional file 6: Figure S4) [39]. To determine the effects of GR-selective liganding on mutant ER chromatin binding at these enhancer regions, we performed directed GR and mutant ER (HA-ER) ChIP in MCF-7 Y537S ER cells. For *CCND1*, GR bound to the same regulatory region in mutant ER-expressing cells as in wild-type ER cells with chromatin association peaking at 30 min following Dex and C335 treatment (Fig. 5a, left panel). Similarly, GR bound to the *CDK2* enhancer region and chromatin association peaked at 30 min following Dex and C335 treatment (Fig. 5a, middle panel). At the *CDK6* enhancer, GR liganding resulted in GR chromatin association peaking at 15 min following Dex, 30 min following C134, and 60 min following C335 treatment (Fig. 5a, right panel). Mutant ER binding was more variable (Fig. 5b). For the *CCND1* enhancer, mutant ER binding was reduced with all GR ligands at 60 min, while *CDK2* and *CDK6* enhancer occupancy was not affected (Fig. 5b). Taken together, these results suggest that GR-selective liganding is associated with suppression of mutant (Y537S) ER chromatin association at the *CCND1* enhancer; while *CDK2* and *CDK6* gene expression downstream of GR activation occurs, it appears be through a mechanism that does not involve loss of mutant ER by 60 min.

### SGRMs inhibit mutant ER (Y537S) MCF-7 tumor growth in vivo

**Fig. 6:**
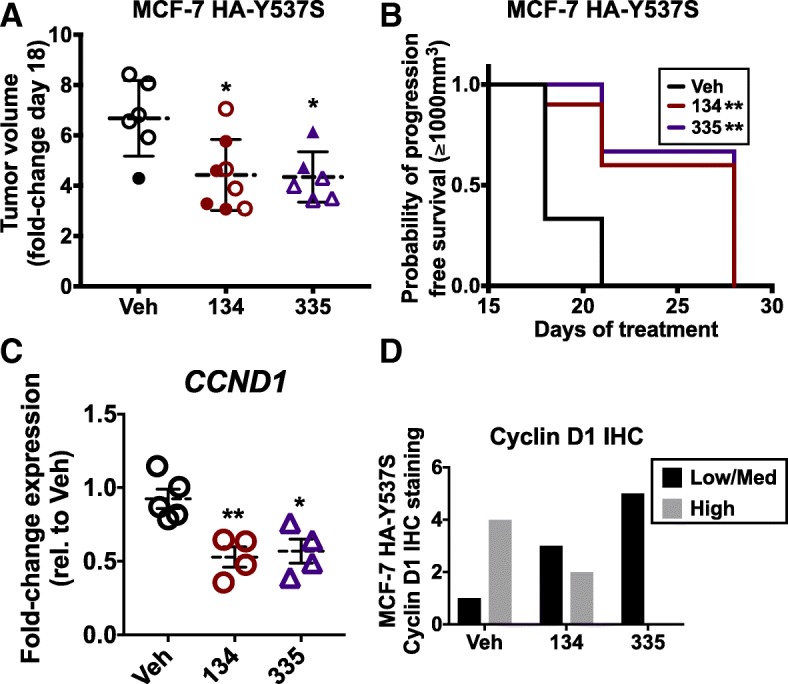
SGRM treatment inhibits MCF-7 Y537S xenograft growth in vivo and is associated with decreased tumor *CCND1* expression. MCF-7 HA-Y537S cells xenografted into SCID mice were treated 3 times weekly with 20 mg/kg C134 (134), 20 mg/kg C335 (335), or vehicle (Veh, 1 ETOH:9 sesame oil) for the duration of the study. **a** After 18 days of treatment with C134 or C335, there was significantly less tumor growth compared to vehicle-treated mice (*p < 0.05, one-way ANOVA, Tukey’s post hoc test, *n* = 6–8 per group, ±SD). Open circles represent tumors analyzed for *CCND1* gene expression. **b** Median progression-free survival following vehicle, C134 or C335 was 18, 28, and 28 days, respectively (**p < 0.003, Log-rank (Mantel-Cox) test, *n* = 6–8 per group). **c** RNA was isolated from tumors, and *CCND1* mRNA expression was measured. Compared to Veh-treated mice, *CCND1* mRNA levels were significantly reduced following treatment with either C134 or C335 (*p < 0.05, **p < 0.01; vs. Veh, two-way ANOVA, Tukey’s post hoc test, *n* = 4–5 per group, ±SD). **d** MCF-7 HA-Y537S xenograft tumors were examined for Cyclin D1 IHC. Tumors from C335-treated mice had significantly lower Cyclin D1 IHC intensity compared to vehicle-treated mice (*p* = 0.0476, Fisher’s exact test, *n* = 5 per group). More C134-treated tumors (3/5) than vehicle (1/5) had low-intensity staining, but the difference did not reach statistical significance

Having discovered that GR liganding suppresses mutant HA-Y537S ER chromatin association with the *CCND1* enhancer, we next wished to determine whether SGRM treatment could also reduce mutant Y537S ER tumor growth in vivo. SCID mice were fed doxycycline-containing chow for 1 week prior to implantation of MCF-7 HA-Y537S cells in the mammary gland as previously described [16]. When tumors reached ~ 200 mm^3^ (Additional file 7: Figure S5A), mice were treated with vehicle, 20 mg/kg C134 or C335 thrice weekly. After 18 days of SGRM treatment (when vehicle-treated control animals had achieved maximally allowed tumor growth), we measured tumor volumes in C134 and C335 SGRM-treated mice (Fig. 6a). We found a significant decrease in average tumor volumes of SGRM-treated animals suggesting decreased cell proliferation of activating mutant ER tumor cells (Fig. 5a). In addition, tumor growth over time was significantly slowed in C134- and C335-treated mice compared to vehicle-treated mice (Additional file 7: Figure S5B). There were no overt signs of animal toxicity during SGRM treatment and body weights remaining relatively stable (Additional file 7: Figure S5C-D). SGRMs also led to significantly increased progression-free survival times (PFS) as defined by tumor volume reaching > 1000 mm^3^ (28 days for SGRMs versus 18 days for control animals, Fig. 6b). All tumor treatments resulted in similarly strong tumor ER intensity and percentage positivity by IHC staining (Additional file 8: Figure S6A, C). Tumor RNA isolation showed significantly decreased steady-state expression of *CCND1* following either C134 (*p* = 0.0055) or C335 treatment (*p* = 0.0105, Fig. 6c). While IHC analysis revealed that all tumors had > 90% of cells with some Cyclin D1 positivity, staining intensity differed between treatment groups. In C335-treated mice, 5/5 tumors exhibited significantly lower intensity Cyclin D1 staining compared to vehicle-treated mice (*p* = 0.0476, Fig. 6D and Additional file 8: Figure S6C). For C134-treated mice, Cyclin D1 IHC staining was of relatively low intensity in 3/5 tumors (*p* value comparison with vehicle-treated tumors (1/5) was not statistically significant *p* = 0.52, Fig. 6d). Tumors from C134- and C335-treated mice also showed a trend toward a decreased percentage of Ki-67-positive cells (*p* = 0.384 for C134 vs. vehicle and *p* = 0.142 for C335 vs vehicle, Additional file 8: Figure S6B-C). Taken together, these data demonstrate that GR-selective liganding can decrease activating mutant (Y537S) ER tumor growth in association with reduced tumor *CCND1* mRNA expression.

## Discussion

Identifying novel methods that decrease ER oncogenic activity and reduce BC progression is a critically important clinical problem. Modulation of global ER transcriptional activity by other nuclear receptors in ER+ BC has now been described by several groups [1–3, 6, 7, 9, 43, 44]. ER activity appears to be significantly regulated by the liganding of non-ER nuclear hormone receptors (i.e., nuclear receptor “crosstalk”) and resultant chromatin modulation. For example, we previously showed that GR and ER could interact on chromatin at transcriptional regulatory regions of certain pro-differentiating ER target genes (*IGFBP4*, *KDM4B*, and *VDR*); in those cases, GR did not diminish ER enhancer association. In fact, ER/GR co-localization increased the expression of these ER target genes compared to ER-activation alone [9]. The Mancini group showed that ER/GR co-localization can either increase or decrease gene expression in a gene context-dependent way [6]. The Rosenfeld group detailed GR and ER association at *TFF1* and *FOXC1* enhancer regions and showed that activated GR prevented cooperating transcription factors (RARα, GATA3, AP2γ, and p300 members of the MegaTrans complex) from binding to these specific ER-targeted enhancers thereby decreasing gene expression [3]. None of these studies examined the mechanism by which GR activation might inhibit gene expression of critical cell cycle genes associated with BC progression (e.g., *CCND1* and *CDK6* genes). Here, we examined the link between GR activity and ER-mediated cell cycle gene expression. We found that both GR agonists and antagonists promote GR chromatin association at several regions of enhancer DNA where they suppress ER occupancy of critical genes that drive ER-mediated cell proliferation.

Our current working paradigm is that SGRM-liganded GR remodels specific enhancer regions, changing their chromatin  conformation and making them less accessible for ER occupancy. How transcription factors, including GR, remodel chromatin to affect ER access to key ER-targeted enhancer regions is a fundamentally important question in cancer biology [45]. Recent work from the Hager lab using single-cell live imaging showed that GR has a highly dynamic association with chromatin [14, 45]. Although we investigated cell populations rather than observing single-cell GR chromatin association, we found a time-dependent and relatively early effect—i.e., 15 and 30 min, on peak SGRM-induced GR occupancy of ER-targeted enhancer regions. While the mechanism by which SGRM-liganded GR can decrease subsequent ER chromatin association (60 min) at specific proliferative gene enhancers is presumed to involve stoichiometric competition between ER and GR, further technical advances in single-cell live imaging and single-cell ChIP will be required to confirm this hypothesis.

Overall, our findings point to a potentially critical and targetable role for GR in regulating proliferative gene expression in ER+ BC cells. Interestingly, examination of previously performed PR ChIP-seq in MCF-7 cells does not reveal PR binding at these same ER-targeted enhancer regions [1]. Furthermore, T-47D cells, which have significantly more PR than GR expression, do have some activated PR binding at the *CDK2* and *CDK6* ER-targeted enhancer regions, but do not demonstrate any PR occupancy at the *CCND1* enhancer [46]. Our findings reveal that GR-selective liganding suppresses Y537S ER chromatin association at the *CCND1* enhancer, inhibits mutant (Y537S) MCF-7 proliferation, and slows Y537S ER+ tumor growth. To our knowledge, this is the first in vivo demonstration of suppression of mutant ER+ BC growth via modulation of a non-ER nuclear receptor.

## Conclusions

GR activation has previously been associated with decreased ER+ BC cell proliferation, although the molecular mechanisms are not well understood. Here, we find that GR-selective liganding decreases ER occupancy of well-characterized ER-target gene (*CCND1*, *CDK2*, and *CDK6*) enhancer regions [21, 33–37]. These findings point to a potentially critical role for GR in regulating pro-proliferative ER-mediated gene expression and support a paradigm in which employing alternative nuclear receptor modulation for endocrine therapy-resistant ER+ BC can be considered. Unlike pure synthetic steroidal agonists (Dex), SGRMs appear to be relatively well-tolerated [47] and are without typical metabolic side effects. Further work toward understanding the mechanisms of suppressing ER-driven cancer by GR modulation is warranted.

## Additional files

Additional file 1: Supplementary information [20, 48–52]. (DOCX 22 kb)

Additional file 2: Supplemental data. MCF-7 cells were treated with Vehicle (Veh, ETOH), 10 nM estradiol (E2), 100 nM Dexamethasone (Dex), or Dex/E2 for 4 h, RNA was isolated and examined by microarray. Microarray fold-changes were calculated relative to Veh and a 1.3 fold-change cut-off was used. (XLSX 255 kb)

Additional file 3: **Figure S1.** SGRM treatment-alone does not affect cell survival in ER+/GR+/PR+ tumor cells. A) Steady-state PR, GR, ER, and β-actin protein expression was evaluated in ER+/GR+ MCF-7 and T-47D cells. B) MCF-7 cells were treated with vehicle (ETOH), 10 nM E2, E2/100 nM Dex, E2/1 μM C134, or E2/1 μM C335 for 2, 4, or 6 h. Steady-state mRNA expression of canonical GR target gene, *FKBP5*, was significantly repressed following C134 or C335 treatment ($ *p* < 0.05 Dex/E2 vs. Veh; *p < 0.05 Dex/E2 vs. C134/E2; **p < 0.005 Dex/E2 vs. C335/E2; ***p < 0.001 Dex/E2 vs. C335/E2; ****p < 0.0001 Dex/E2 vs. C134/E2; two-way ANOVA, Tukey’s post hoc test, *n* = 3 per group, ±SD). C) MCF-7 and T-47D cell death measure by trypan blue exclusion over 8 days following vehicle (ETOH), 10 nM E2, E2/100 nM Dex, E2/1 μM C134, or E2/1 μM C335 treatment (NS, not significant; two-way ANOVA, Tukey’s post hoc test). (PDF 255 kb)

Additional file 4: **Figure S2.** SGRMs do not effect ER-mediated T47D-Y (GR-negative) cell proliferation or bind to purified ER LBD. A) Steady-state GR, ER-α, and β-actin expression was evaluated in parental T47-D and ER+/GR-negative T47D-Y cells. B) T47D-Y cell proliferation during 8 days vehicle (ETOH, Veh), 10 nM E2, E2/100 nM Dex, E2/1 μM C297, or E2/1 μM C335 treatment. GR liganding (Dex, C297, or C335) does not inhibit E2-mediated proliferation (****p < 0.0001 vs. vehicle; NS, not significant; two-way ANOVA, Tukey’s post hoc test, *n* = 3 per group, ±SD). C) Purified ER ligand binding domain (LBD) (5 nM) was incubated with 10 nM tritiated (H3)-E2 and increasing concentrations (0.1–10,000 nM) of Dex, C134, and C335 or E2 for 30 min. GR ligands do not competitively bind ER LBD while E2 demonstrates competitive binding to ER LBD. (PDF 160 kb)

Additional file 5: **Figure S3.** GR liganding inhibits MCF-7 mutant ER-driven proliferation and decreases proliferative gene expression. A) Doxycycline induced expression of HA-tagged ER, and GR expression remains stable in all cell lines. B) MCF-7 HA-WT, HA-Y537S, and HA-D538G cells were treated with vehicle (ETOH) and proliferation as evaluated at 6 days of treatment. HA-D538G and HA-Y537S expressing MCF-7 cells show 30–50% increase in cell proliferation, respectively, compared to HA-WT MCF-7 cells. C) Dex, C134 and C335 all significantly inhibited E2-mediated proliferation in both HA-wild type (HA-WT) and HA-D538G MCF-7 cells following 6 days of treatment (*p < 0.05, **p < 0.01, ***p < 0.001, vs. Veh, one-way ANOVA, Tukey’s post hoc test, *n* = 3 per group ±SD). D) MCF-7 HA-D538G cells were treated with vehicle (ETOH), 100 nM Dex, 1 μM C134, or 1 μM C335 for 4 h and mRNA expression was evaluated. *CCND1*, *CDK2*, and *CDK6* gene expression was significantly inhibited by C134 for all genes, by C335 for *CDK2*, and by Dex for *CCND1* and *CDK2* (*p < 0.05; **p < 0.01; ****p < 0.0001; ns, not significant; vs. vehicle; one-way ANOVA, Tukey’s post hoc test, *n* = 3 per group ±SD). (PDF 485 kb)

Additional file 6: **Figure S4.** Mutant Y573S ER and E2 stimulated wild-type ER bind to the same enhancer regions of pro-proliferative genes. MCF-7 cells expressing wild-type ER or mutant Y537S ER demonstrates overlapping chromatin enrichment by ChIP sequencing [39] at CCND1, CDK2 and CDK6 enhancer regions following vehicle (ETOH), 100 nM Dex, 100 nM E2, or Dex/E2 (DE) at 60 min. (PDF 142 kb)

Additional file 7: **Figure S5.** SGRM treatment inhibits ER-mediated tumor growth with minimal toxicity in association with decreased ER-mediated pro-proliferative gene expression. A) Average tumor volume in MCF-7 HA-Y537S mouse xenografts in each treatment group, vehicle (Veh), C134 (134), and C335 (335) at the start of treatment. B) Longitudinal tumor growth following vehicle (Veh, 1 ETOH:9 sesame oil), 20 mg/kg C134 (134), or 20 mg/kg C335 (335) (**p < 0.01, ***p < 0.001, vs. Veh, repeated measures two-way ANOVA, Dunnett’s post hoc test, *n* = 6–8 per group, ±SEM). C) Longitudinal body weights (*p* = 0.9037, vs. vehicle, two-way ANOVA, Tukey’s post hoc test, *n* = 6–8 per group). D) Scatter plot of body weights at day 0 and 18 of treatment (*p* = 0.6979, vs. vehicle, two-way ANOVA, Holm Sidak post hoc test, *n* = 6–8 per group). (PDF 85 kb)

Additional file 8: **Figure S6.** MCF-7 Y537S xenograft tumor IHC shows decreased Ki-67 positivity and no change in ER expression following SGRM treatment. A) Anti-ERα IHC staining (H-score) showed no difference among vehicle (Veh), C134 (134), or C335 (335) treatment groups (*p* = 0.931, One-way ANOVA, Dunnett’s post hoc test, *n* = 5 per group). B) Anti-Ki-67 IHC staining. Tumors treated in vivo with C134 or C335 showed a decreased trend in Ki-67 percentage compared to vehicle treatment (*p* = 0.384, Veh vs 134; *p* = 0.142, Veh vs 335; one-way ANOVA, Dunnett’s post hoc test, *n* = 5 per group). C) Representative images of anti-ERα, anti-Ki-67, and anti-Cyclin D1 IHC immunostaining. Scale bar shown in red is 100 μm. (PDF 618 kb)

## Data Availability

The ER ChIP-seq and global gene expression datasets generated during the current study will be available in NCBI Gene Expression Omnibus (GEO) repository. The datasets analyzed during the current study are available in the GEO repository, https://www.ncbi.nlm.nih.gov/geo/query/acc.cgi?acc=GSE41324 [36]; https://www.ncbi.nlm.nih.gov/geo/query/acc.cgi?acc=GSE45822 [37]; https://www.ncbi.nlm.nih.gov/geo/query/acc.cgi?acc=GSE78286 [39]; https://www.ncbi.nlm.nih.gov/geo/query/acc.cgi?acc=GSE94493 [42].

## Abbreviations

ANOVA: Analysis of variance
AP2γ: Activating enhancer-binding protein 2 alpha
AR: Androgen receptor
ATCC: American tissue culture collection
BC: Breast cancer
BCA: Bicinchoninic acid
BSA: Bovine serum albumin
C134: CORT125134
C297: CORT108297
C335: CORT118335
CCDN1: Cyclin D1
CDK2: Cyclin-dependent kinase 2
CDK6: Cyclin-dependent kinase 6
cDNA: Complementary deoxyribonucleic acid
ChIP: Chromatin immunoprecipitation
ChIP-seq: Chromatin immunoprecipitation sequencing
CO_2_: Carbon dioxide
CPG: Controlled Pore Glass
CSS: Charcoal-stripped serum
DE: Dexamethasone/Estradiol
Dex: Dexamethasone
DMEM: Dulbecco’s modified Eagle’s medium
DNA: Deoxyribonucleic acid
Dox: Doxycycline
E2: Estradiol
Enh2: Enhancer 2
ER: Estrogen receptor
ER +: Estrogen receptor-positive
ERE: Estrogen receptor response element
eRNA: Enhancer RNA
ETOH: Ethanol
FACS: Fluorescence-activated cell sorting
FBS: Fetal bovine serum
FKBP5: FK506 binding protein 5
FOXA1: Forkhead box protein A1
FOXC1: Forkhead box C1
GATA3: GATA binding protein 3
GFP: Green fluorescent protein
GR: Glucocorticoid receptor
GRE: Glucocorticoid receptor response element
H3: Tritiated
HA: human influenza hemagglutinin
IgG: Immunoglobulin G
i.p.: Intraperitoneal
IGFBP4: Insulin-like growth factor binding protein 4
IHC: Immunohistochemistry
IPA: Ingenuity Pathway Analysis
LBD: Ligand-binding domain
KDM4B: Lysine demethylase 4B
Ki-67: MKI67
mRNA: Messenger ribonucleic acid
MSKCC: Memorial Sloan Kettering Cancer Center
NS: Not significant
p300: Histone acetyltransferase p300
PFS: Progression-free survival
PR: Progesterone receptor
qPCR: Quantitative polymerase chain reaction
qRT-PCR: Quantitative reverse transcription polymerase chain reaction
RARα: Retinoic acid receptor alpha
RE: Response element
RIPA: Radioimmunoprecipitation assay
RPLP0: Ribosomal Protein Lateral Stalk Subunit P0
RNA: Ribonucleic acid
RT: Room temperature
SCID: Severe combined immunodeficiency
SD: Standard deviation
SDS-PAGE: Sodium dodecyl sulfate polyacrylamide gel electrophoresis
SEM: Standard error of the mean
SGRM: Selective GR modulator
NR: Nuclear receptor
TF: Transcription factor
TSS: Transcription start site
TFF1: Trefoil factor 1
VDR: Vitamin D receptor
Veh: Vehicle
vs.: Versus
