# Supplementary material for: Glucocorticoid receptor modulation decreases ER-positive breast cancer cell proliferation and suppresses wild-type and mutant ER chromatin association
Source: Breast Cancer Res. 2019 Jul 24;21:82. doi: 10.1186/s13058-019-1164-6 (PMC6651939; doi:10.1186/s13058-019-1164-6)
Supplement: Supplementary file 1 — Supplementary information [20, 48–52]. (DOCX 22 kb) [file 13058_2019_1164_MOESM1_ESM.docx]

**Supplementary Materials and Methods**

**ER LBD Displacement Assay.** 5 nM purified ER LBD protein was incubated with 10 nM tritiated (H3)-E2 and with increasing concentrations (0.1-10,000 nM) of GR ligand (Dex, C134, or C335) or E2 for 30 minutes at RT. Reaction mixtures were added to Controlled Pore Glass (CPG) bead columns to capture H3-E2-bound ER LBD. Free H3-E2 was washed away with 10 column volumes of wash buffer (10 mM Tris pH 7.4, 400 mM NaCl). Captured H3-E2-bound ER LBD was eluted with 1 mL ethanol and radioactivity was measured with a Packard Tri-Carb 2200CA Liquid Scintillation Counter.

**Bioinformatics Analyses.** *Microarray processing* - CEL files were processed, normalized (RMA), and converted to expression sets in R with packages limma (3.32.10), annaffy (1.48.0), affy (1.54.0), and hgu133plus2hsentrezg.db (20.0.0). Genes were filtered using a log2 fold-change cutoff of 1.3 (relative to vehicle).

*ChIP-seq processing* – Raw reads were trimmed and filtered using Trimmomatic v0.33 (*parameters*: ILLUMINACLIP:TruSeq3-SE.fa:2:30:10 LEADING:3 TRAILING:3 SLIDINGWINDOW:4:15 MINLEN:36; [48]. Processed reads were aligned to hg19 using BWA aln v0.7.12 [49] and post-processed (filtered, merged, and PCR duplicate marking) with sambamba v0.5.9 (*filtering parameters: -F "mapping_quality >= 20 and not unmapped"*; [50]. Peaks were detected using MACS2 v2.1.1.20160309 [20]. Peaks were annotated using homer v4.5 [51]. Peaks with an unadjusted P-value greater than 0.0001 were removed from further analysis. Filtered peaks were considered to be associated with a gene if they were within 100 kb of the TSS. Bigwig files (relative to input) and tag heatmap figures were generated using deepTools [52].

**qRT-PCR Primers**.

| Gene | Forward | Reverse |
| --- | --- | --- |
| *CCND1* | 5’- TCTACACCGACAACTCCATCCG -3’ | 5’- TCTGGCATTTTGGAGAGGAAGTG -3’ |
| *CDK2* | 5’- ATGGATGCCTCTGCTCTCACTG-3’ | 5’- CCCGATGAGAATGGCAGAAAGC-3’ |
| *CDK6* | 5’-GGATAAAGTTCCAGAGCCTGGAG-3’ | 5’-GCGATGCACTACTCGGTGTGAA-3’ |
| *FKBP5* | 5'-CTGAAGGGTTAGCGGAGCA-3' | 5'-CTGTGGGGCTTTCTTCATTG-3' |
| *RPLP0* | 5'-GGAGAAACTGCTGCCTCATATC-3' | 5'-CAGCAGCTGGCACCTTATT-3' |
