# Supplementary figures and images for: Glucocorticoid receptor modulation decreases ER-positive breast cancer cell proliferation and suppresses wild-type and mutant ER chromatin association
Source: Breast Cancer Res. 2019 Jul 24;21:82. doi: 10.1186/s13058-019-1164-6 (PMC6651939; doi:10.1186/s13058-019-1164-6)

**A**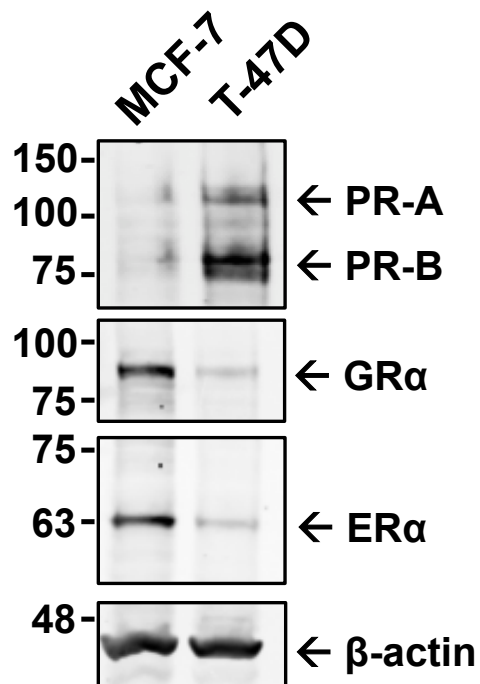**B**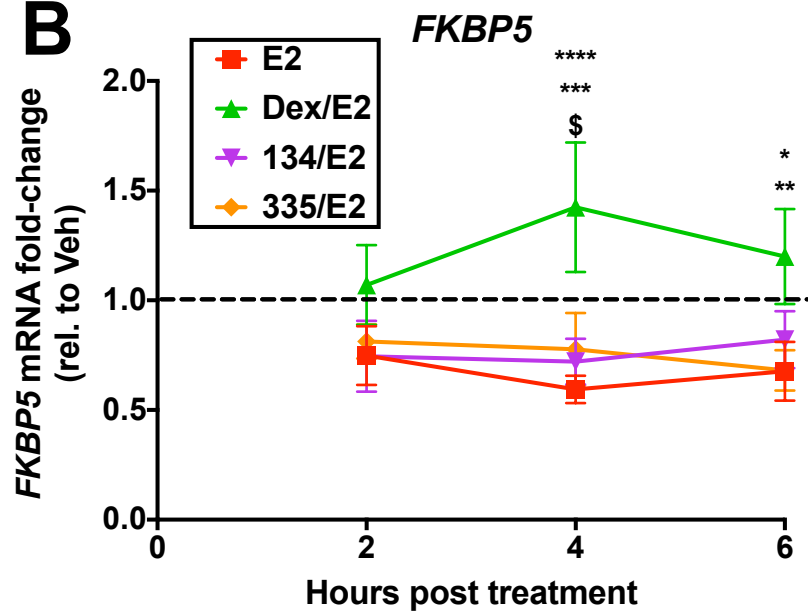**C**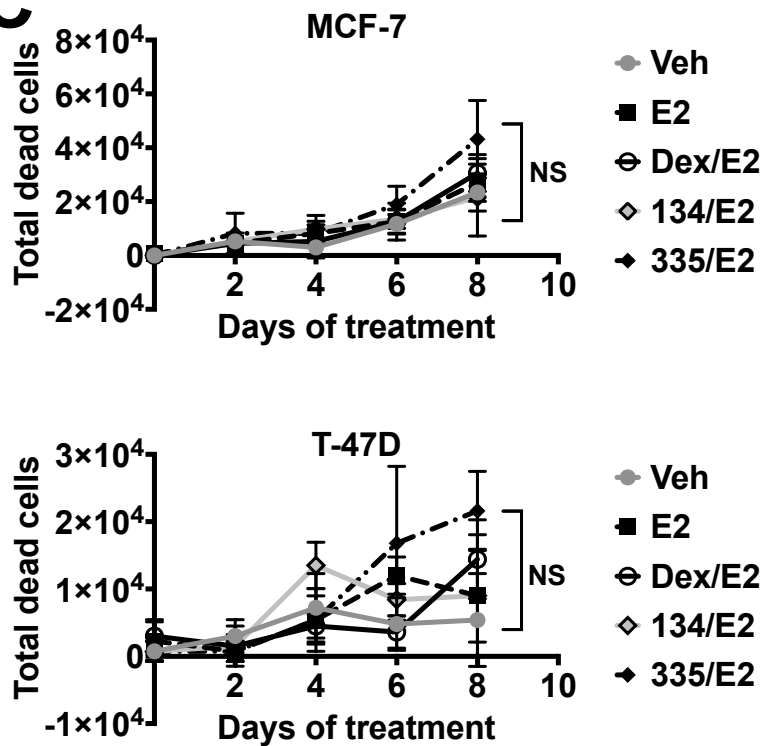

Supplement: Supplementary file 3 — Figure S1. SGRM treatment-alone does not affect cell survival in ER+/GR+/PR+ tumor cells. A) Steady-state PR, GR, ER, and β-actin protein expression was evaluated in ER+/GR+ MCF-7 and T-47D cells. B) MCF-7 cells were treated with vehicle (ETOH), 10 nM E2, E2/100 nM Dex, E2/1 μM C134, or E2/1 μM C335 for 2, 4, or 6 h. Steady-state mRNA expression of canonical GR target gene, FKBP5, was significantly repressed following C134 or C335 treatment ($ p < 0.05 Dex/E2 vs. Veh; *p < 0.05 Dex/E2 vs. C134/E2; **p < 0.005 Dex/E2 vs. C335/E2; ***p < 0.001 Dex/E2 vs. C335/E2; ****p < 0.0001 Dex/E2 vs. C134/E2; two-way ANOVA, Tukey’s post hoc test, n = 3 per group, ±SD). C) MCF-7 and T-47D cell death measure by trypan blue exclusion over 8 days following vehicle (ETOH), 10 nM E2, E2/100 nM Dex, E2/1 μM C134, or E2/1 μM C335 treatment (NS, not significant; two-way ANOVA, Tukey’s post hoc test). (PDF 255 kb) [file 13058_2019_1164_MOESM3_ESM.pdf]

**A**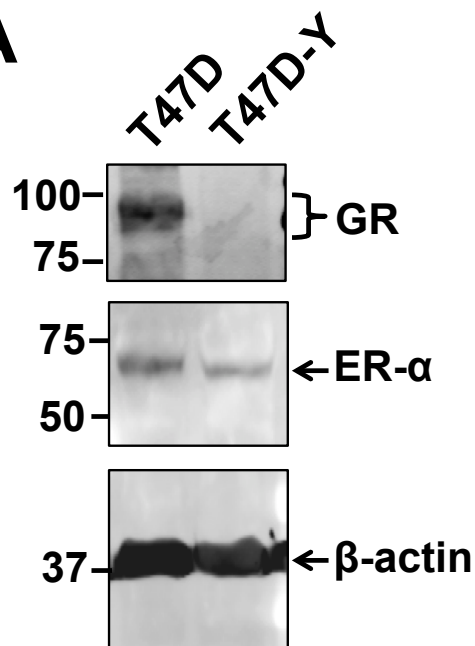**B**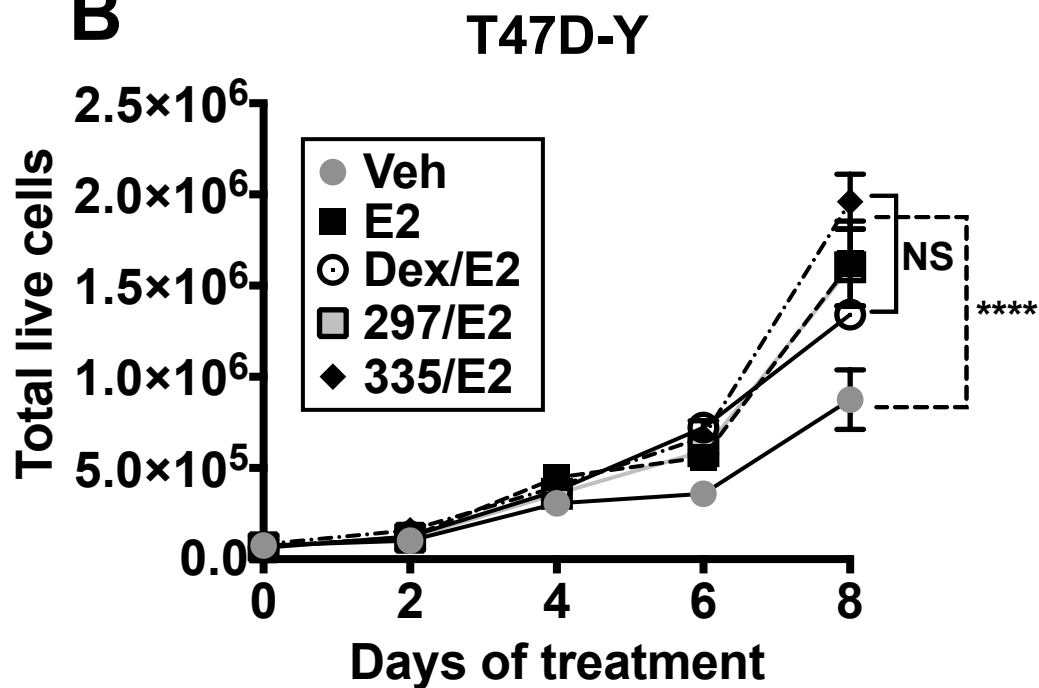**C**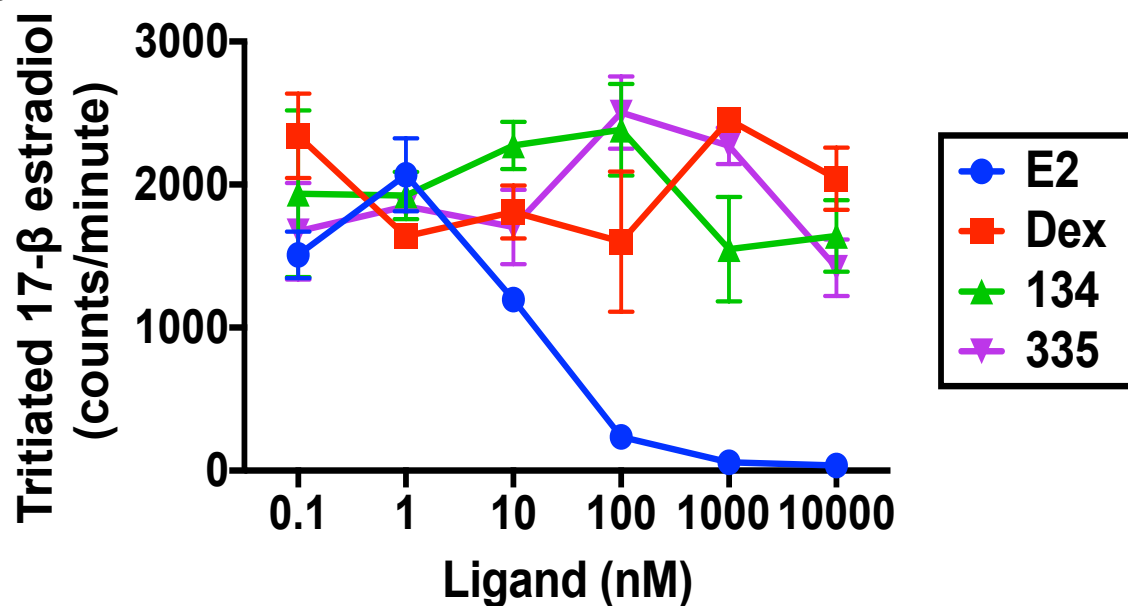

Supplement: Supplementary file 4 — Figure S2. SGRMs do not effect ER-mediated T47D-Y (GR-negative) cell proliferation or bind to purified ER LBD. A) Steady-state GR, ER-α, and β-actin expression was evaluated in parental T47-D and ER+/GR-negative T47D-Y cells. B) T47D-Y cell proliferation during 8 days vehicle (ETOH, Veh), 10 nM E2, E2/100 nM Dex, E2/1 μM C297, or E2/1 μM C335 treatment. GR liganding (Dex, C297, or C335) does not inhibit E2-mediated proliferation (****p < 0.0001 vs. vehicle; NS, not significant; two-way ANOVA, Tukey’s post hoc test, n = 3 per group, ±SD). C) Purified ER ligand binding domain (LBD) (5 nM) was incubated with 10 nM tritiated (H3)-E2 and increasing concentrations (0.1–10,000 nM) of Dex, C134, and C335 or E2 for 30 min. GR ligands do not competitively bind ER LBD while E2 demonstrates competitive binding to ER LBD. (PDF 160 kb) [file 13058_2019_1164_MOESM4_ESM.pdf]

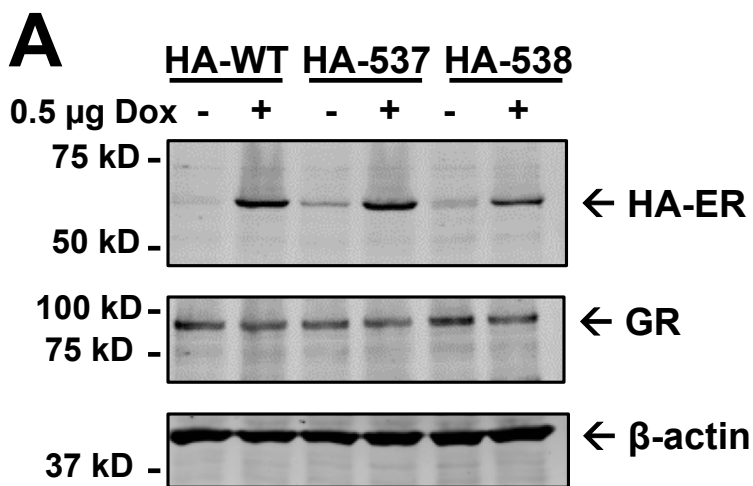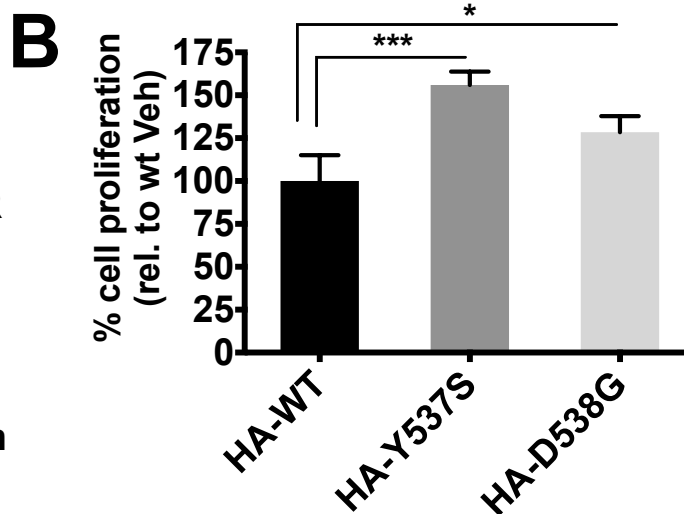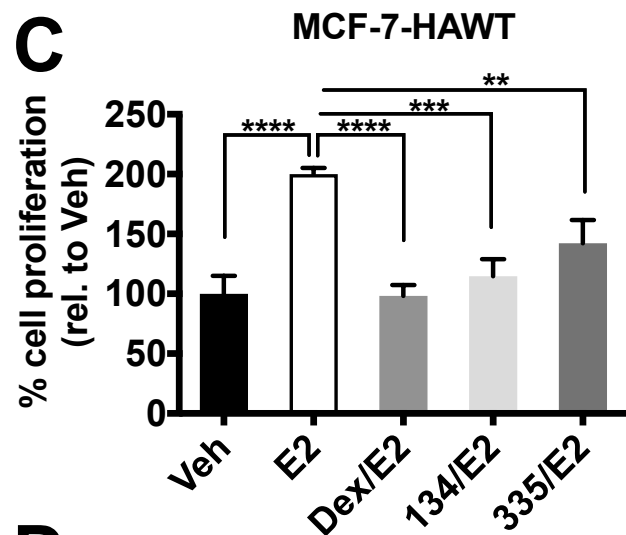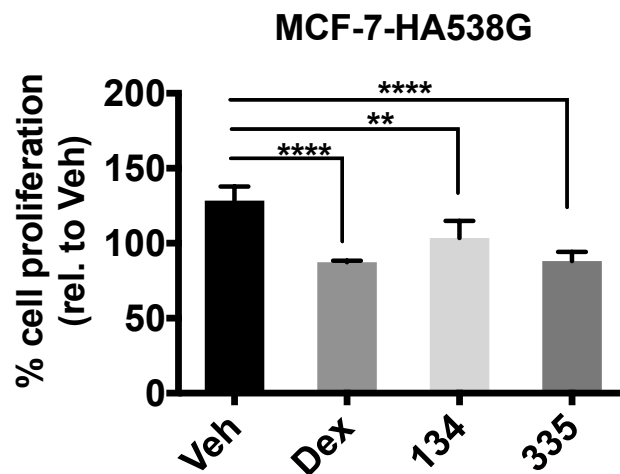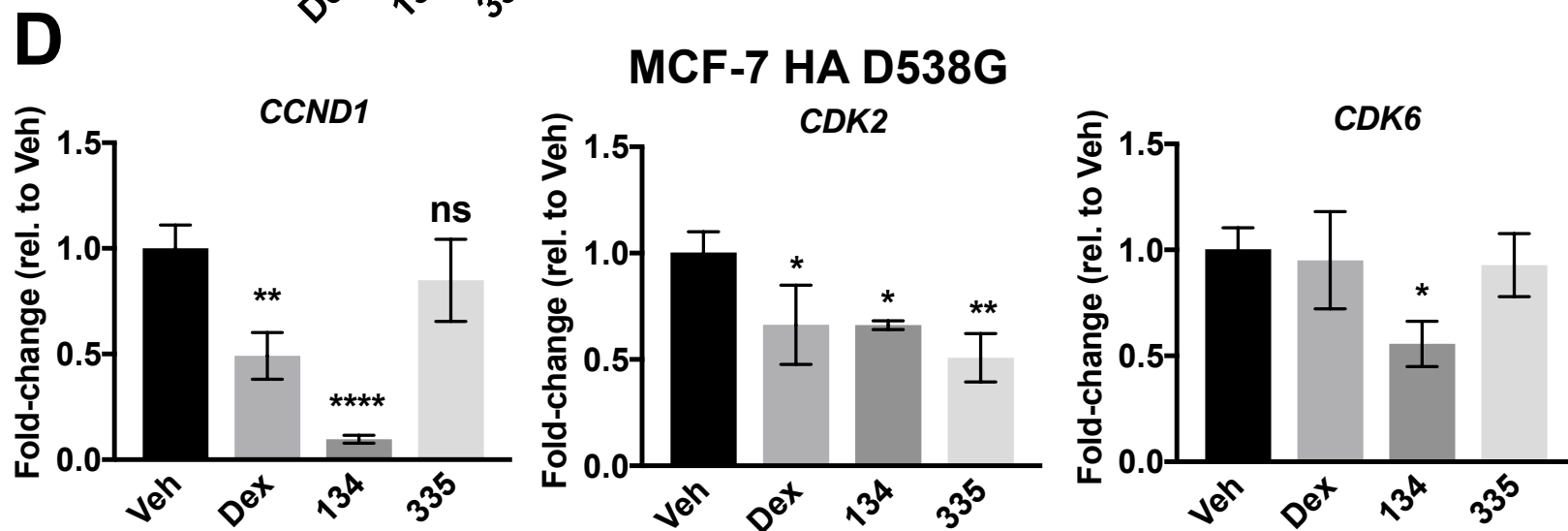

Supplement: Supplementary file 5 — Figure S3. GR liganding inhibits MCF-7 mutant ER-driven proliferation and decreases proliferative gene expression. A) Doxycycline induced expression of HA-tagged ER, and GR expression remains stable in all cell lines. B) MCF-7 HA-WT, HA-Y537S, and HA-D538G cells were treated with vehicle (ETOH) and proliferation as evaluated at 6 days of treatment. HA-D538G and HA-Y537S expressing MCF-7 cells show 30–50% increase in cell proliferation, respectively, compared to HA-WT MCF-7 cells. C) Dex, C134 and C335 all significantly inhibited E2-mediated proliferation in both HA-wild type (HA-WT) and HA-D538G MCF-7 cells following 6 days of treatment (*p < 0.05, **p < 0.01, ***p < 0.001, vs. Veh, one-way ANOVA, Tukey’s post hoc test, n = 3 per group ±SD). D) MCF-7 HA-D538G cells were treated with vehicle (ETOH), 100 nM Dex, 1 μM C134, or 1 μM C335 for 4 h and mRNA expression was evaluated. CCND1, CDK2, and CDK6 gene expression was significantly inhibited by C134 for all genes, by C335 for CDK2, and by Dex for CCND1 and CDK2 (*p < 0.05; **p < 0.01; ****p < 0.0001; ns, not significant; vs. vehicle; one-way ANOVA, Tukey’s post hoc test, n = 3 per group ±SD). (PDF 485 kb) [file 13058_2019_1164_MOESM5_ESM.pdf]

ER Veh  
ER Dex  
ER E2  
ER DE  
Y537S Veh

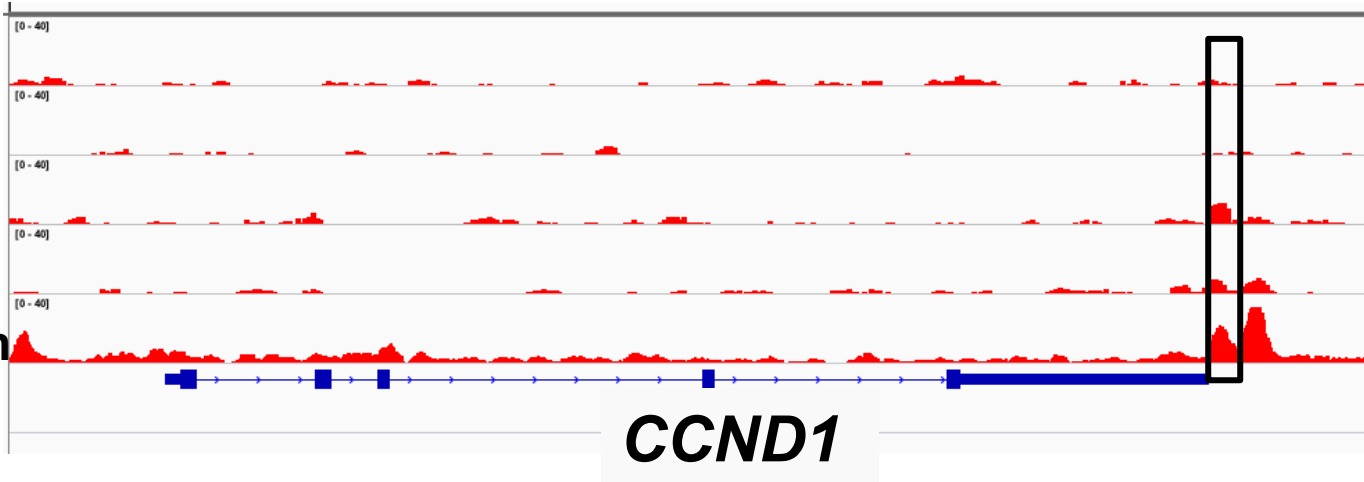

ER Veh  
ER Dex  
ER E2  
ER DE  
Y537S Veh

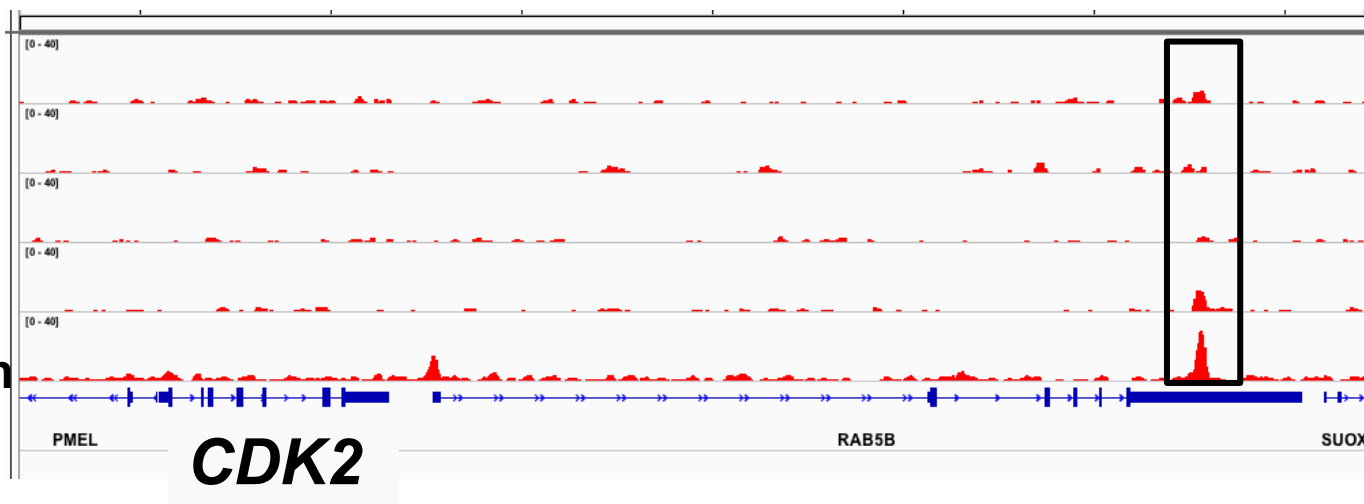

ER Veh  
ER Dex  
ER E2  
ER DE  
Y537S Veh

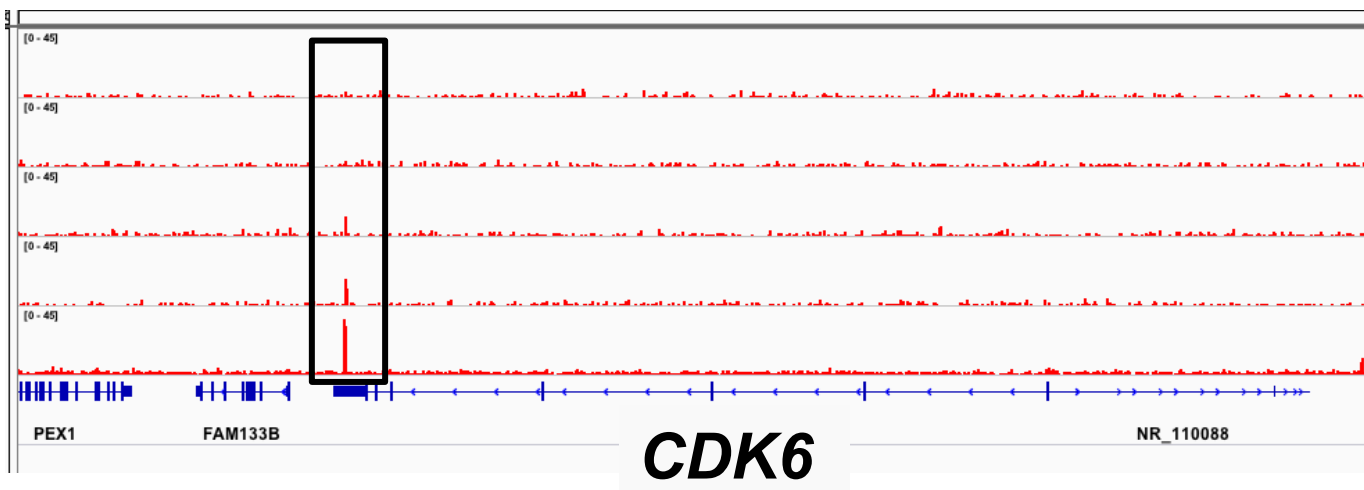

Supplement: Supplementary file 6 — Figure S4. Mutant Y573S ER and E2 stimulated wild-type ER bind to the same enhancer regions of pro-proliferative genes. MCF-7 cells expressing wild-type ER or mutant Y537S ER demonstrates overlapping chromatin enrichment by ChIP sequencing [39] at CCND1, CDK2 and CDK6 enhancer regions following vehicle (ETOH), 100 nM Dex, 100 nM E2, or Dex/E2 (DE) at 60 min. (PDF 142 kb) [file 13058_2019_1164_MOESM6_ESM.pdf]

**A**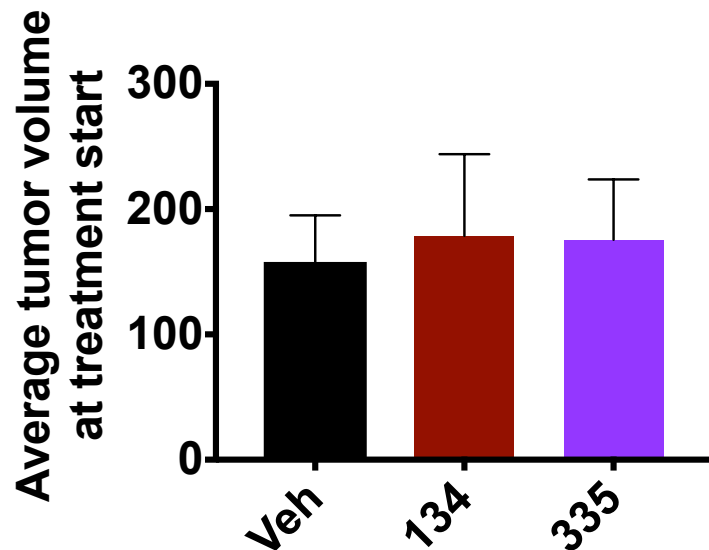**B**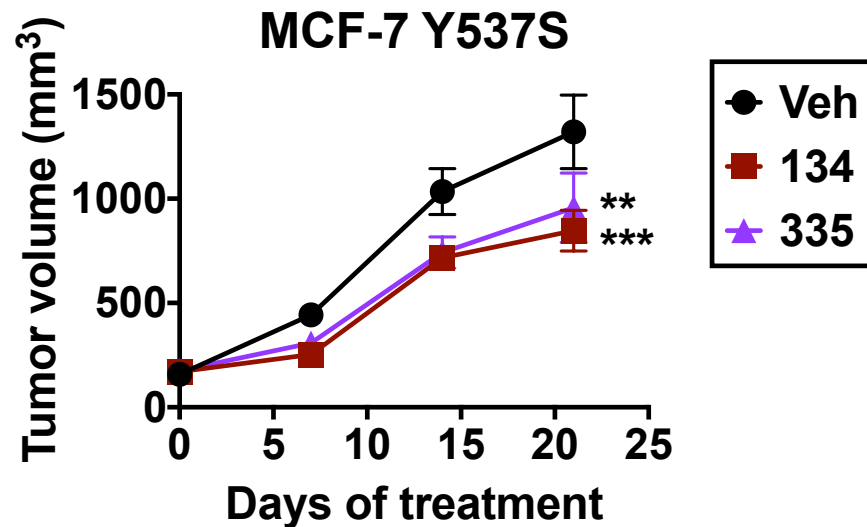**C**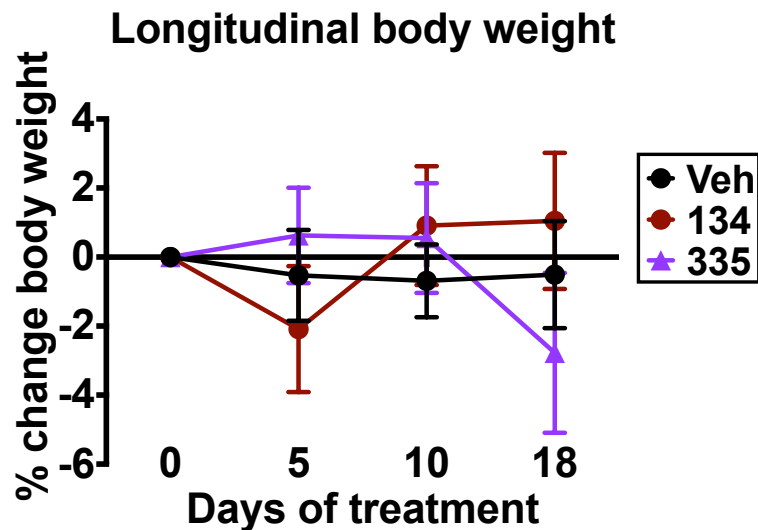**D**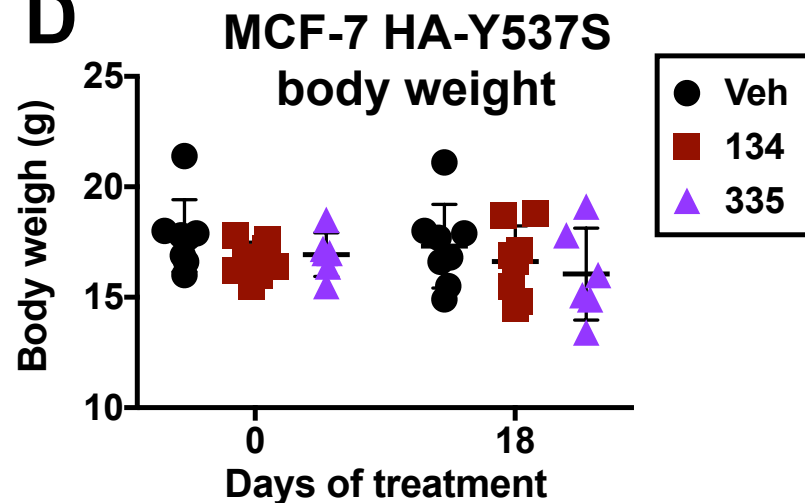

Supplement: Supplementary file 7 — Figure S5. SGRM treatment inhibits ER-mediated tumor growth with minimal toxicity in association with decreased ER-mediated pro-proliferative gene expression. A) Average tumor volume in MCF-7 HA-Y537S mouse xenografts in each treatment group, vehicle (Veh), C134 (134), and C335 (335) at the start of treatment. B) Longitudinal tumor growth following vehicle (Veh, 1 ETOH:9 sesame oil), 20 mg/kg C134 (134), or 20 mg/kg C335 (335) (**p < 0.01, ***p < 0.001, vs. Veh, repeated measures two-way ANOVA, Dunnett’s post hoc test, n = 6–8 per group, ±SEM). C) Longitudinal body weights (p = 0.9037, vs. vehicle, two-way ANOVA, Tukey’s post hoc test, n = 6–8 per group). D) Scatter plot of body weights at day 0 and 18 of treatment (p = 0.6979, vs. vehicle, two-way ANOVA, Holm Sidak post hoc test, n = 6–8 per group). (PDF 85 kb) [file 13058_2019_1164_MOESM7_ESM.pdf]

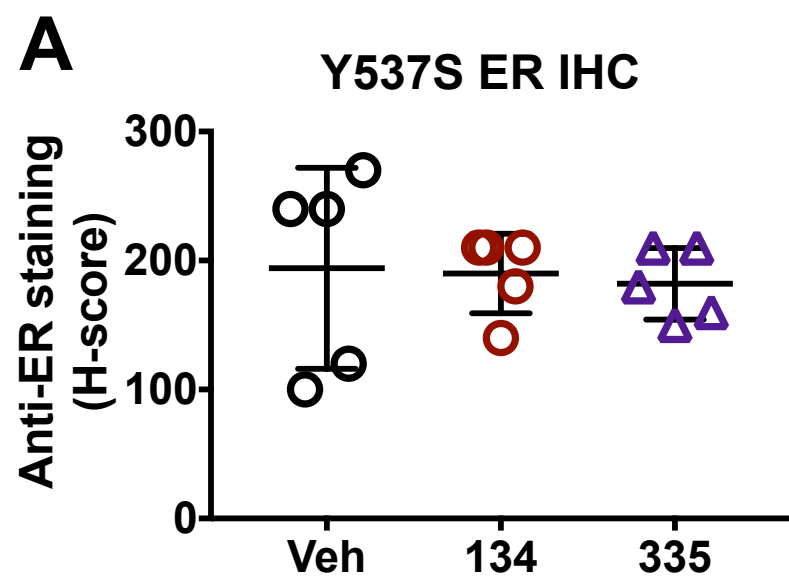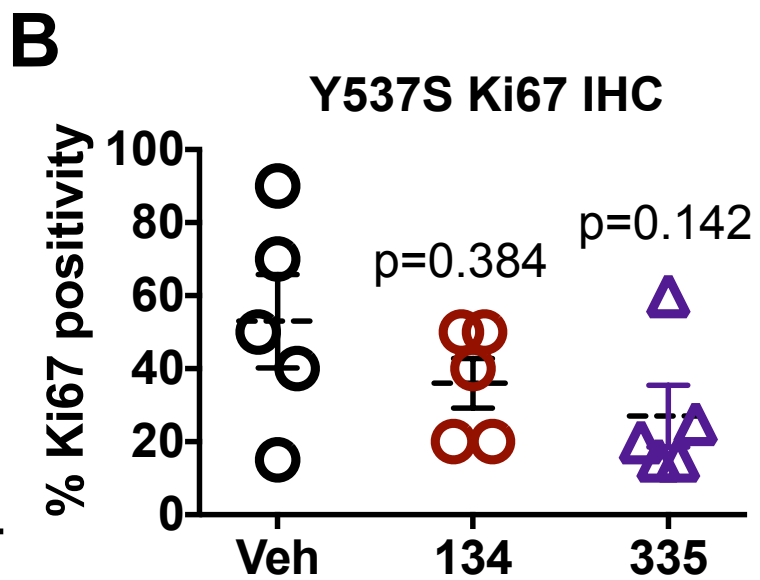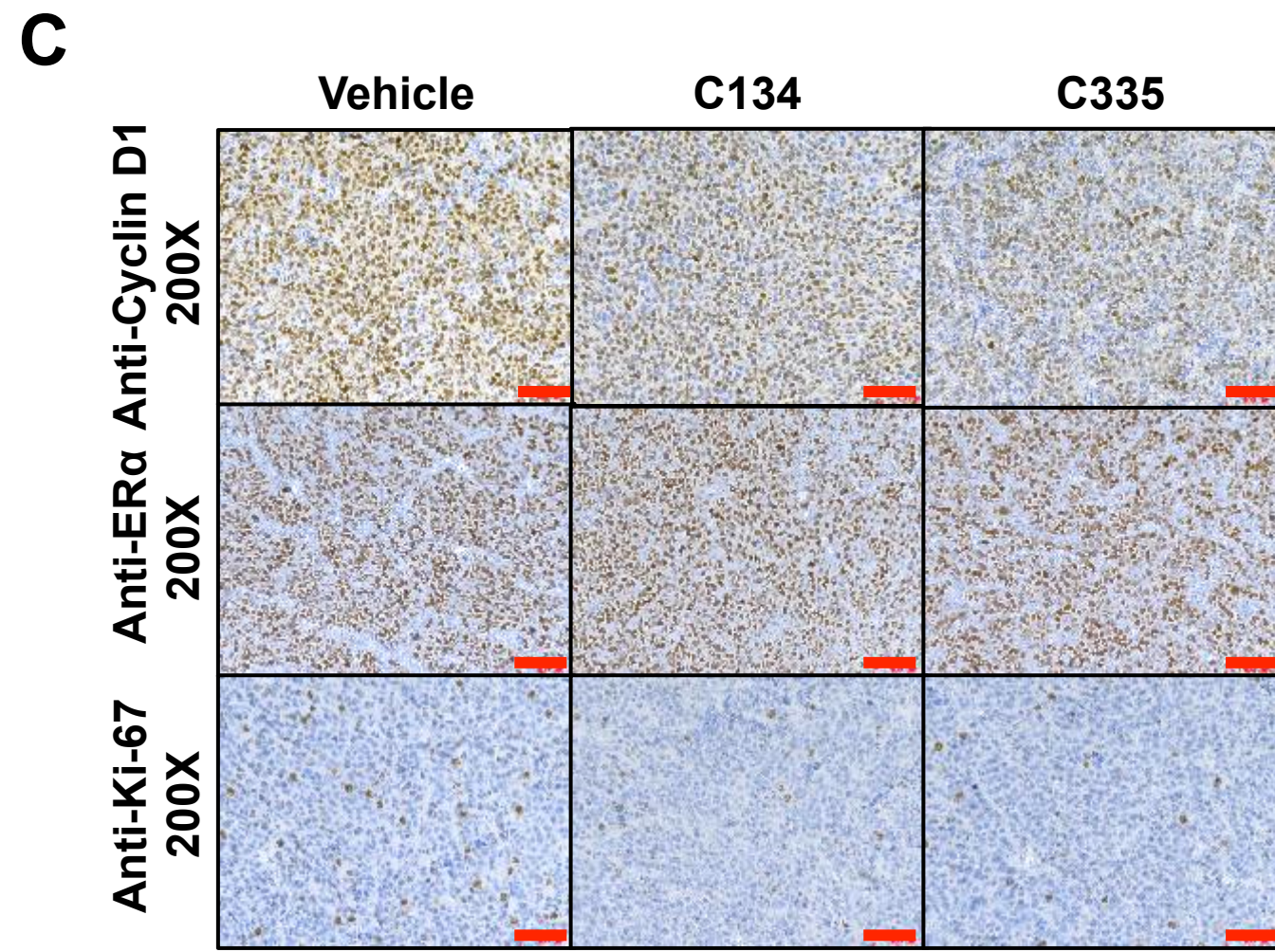

Supplement: Supplementary file 8 — Figure S6. MCF-7 Y537S xenograft tumor IHC shows decreased Ki-67 positivity and no change in ER expression following SGRM treatment. A) Anti-ERα IHC staining (H-score) showed no difference among vehicle (Veh), C134 (134), or C335 (335) treatment groups (p = 0.931, One-way ANOVA, Dunnett’s post hoc test, n = 5 per group). B) Anti-Ki-67 IHC staining. Tumors treated in vivo with C134 or C335 showed a decreased trend in Ki-67 percentage compared to vehicle treatment (p = 0.384, Veh vs 134; p = 0.142, Veh vs 335; one-way ANOVA, Dunnett’s post hoc test, n = 5 per group). C) Representative images of anti-ERα, anti-Ki-67, and anti-Cyclin D1 IHC immunostaining. Scale bar shown in red is 100 μm. (PDF 618 kb) [file 13058_2019_1164_MOESM8_ESM.pdf]
